# Human steroid biosynthesis, metabolism and excretion are differentially reflected by serum and urine steroid metabolomes: A comprehensive review

**DOI:** 10.1016/j.jsbmb.2019.105439

**Published:** 2019-11

**Authors:** Lina Schiffer, Lise Barnard, Elizabeth S. Baranowski, Lorna C. Gilligan, Angela E. Taylor, Wiebke Arlt, Cedric H.L. Shackleton, Karl-Heinz Storbeck

**Affiliations:** a Institute of Metabolism and Systems Research (IMSR), University of Birmingham, Birmingham, UK; b Department of Biochemistry, Stellenbosch University, Stellenbosch, South Africa; c Centre for Endocrinology, Diabetes and Metabolism, Birmingham Health Partners, Birmingham, UK; d Department of Paediatric Endocrinology and Diabetes, Birmingham Women’s and Children’s Hospital NHS Foundation Trust, Birmingham, UK; e NIHR Birmingham Biomedical Research Centre, University Hospitals Birmingham NHS Foundation Trust & University of Birmingham, Birmingham, UK; f UCSF Benioff Children’s Hospital Oakland Research Institute, Oakland, CA, USA

**Keywords:** Steroid metabolome, Steroid biosynthesis, Steroid metabolism, Urine metabolome, Serum metabolome

## Abstract

Advances in technology have allowed for the sensitive, specific, and simultaneous quantitative profiling of steroid precursors, bioactive steroids and inactive metabolites, facilitating comprehensive characterization of the serum and urine steroid metabolomes. The quantification of steroid panels is therefore gaining favor over quantification of single marker metabolites in the clinical and research laboratories. However, although the biochemical pathways for the biosynthesis and metabolism of steroid hormones are now well defined, a gulf still exists between this knowledge and its application to the measured steroid profiles. In this review, we present an overview of steroid hormone biosynthesis and metabolism by the liver and peripheral tissues, specifically highlighting the pathways linking and differentiating the serum and urine steroid metabolomes. A brief overview of the methodology used in steroid profiling is also provided.

## Highlights

• The serum metabolome does not reflect steroids activated in an intracrine manner.
• The urinary steroid metabolome reflects steroid biosynthesis and metabolism.
• Defined pathways link the circulating and urinary steroid metabolomes.
• Modern mass spectrometry techniques allow for comprehensive steroid profiling.

## Graphical abstract

**Figure:**
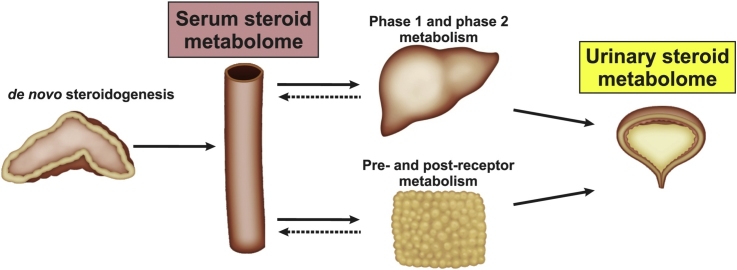

## 1 Introduction

Steroid hormones play an essential role in regulating water and salt balance, metabolism and stress response, and in initiating and maintaining sexual differentiation and reproduction. Researchers investigating steroid-related endocrine conditions have measured alterations in the steroid metabolome for several decades. While clinical laboratories have traditionally measured changes in individual diagnostic marker steroids, the quantification of steroid panels are now gaining widespread traction due to advances in technology, further driven by the emerging diagnostic power of steroid metabolomics, i.e. the combination of mass spectrometry-based steroid profiling with unbiased data analysis by machine learning approaches.

In most cases, alterations in steroid profiles associated with endocrine disorders were identified long before the responsible enzymes were identified or characterized following the advent of modern molecular techniques. While the biochemical pathways for the biosynthesis and metabolism of steroid hormones are now mostly well defined, a gulf still exists with regard to the application of this knowledge to the interpretation of the measured multi-steroid profiles in serum and urine. Researchers and clinicians are increasingly dependent on results obtained by steroid metabolome analysis, but are often unfamiliar with the metabolic pathways resulting in the observed steroid profile and the distinct metabolic pathways explaining the differences between serum and urine steroid metabolomes.

Therefore, it is the aim of this review to provide a comprehensive and up-to-date examination of our current knowledge of metabolic pathways underlying the serum and urine steroid metabolomes. We briefly review the origins of steroid hormones, and present the resulting serum metabolome of each of the main classes of steroids. Downstream metabolism of each of these steroid classes are subsequently presented and linked to the resulting urine steroid excretion patterns. Taken together this review provides a biochemical overview of the biosynthesis, metabolism and excretion of steroid hormones.

## 2 Origins of steroid hormones

### 2.1 Overview of *de novo* steroidogenesis

Steroid hormones are produced through *de novo* steroidogenesis in the adrenal cortex, the gonads and the placenta. In addition, a range of neurosteroids are produced in the brain [1], however these are beyond the scope of this review. Steroidogenic tissues are unique in their ability to utilize cholesterol as starting material for the mitochondrial biosynthesis of pregnenolone, the precursor steroid in the biosynthesis of all steroid hormones. Cholesterol can be obtained from multiple sources including *de novo* biosynthesis from acetate in the endoplasmic reticulum (ER) [[2], [3], [4]], the hydrolysis of cholesteryl esters stored in lipid droplets by cholesteryl ester hydrolases, exogenous lipoprotein-derived cholesterol esters from LDL receptor-mediated endocytic and/or SR-BI-mediated uptake pathways, and free cholesterol residing in the plasma membrane [[5], [6], [7], [8]]. All three primary steroidogenic organs, namely the adrenal cortex, gonads and placenta, can biosynthesize cholesterol *de novo* under the regulation of tropic hormones and plasma lipoproteins are widely accepted as the principal source of cholesterol used for steroid biosynthesis [[5], [6], [7], [8]].

### 2.2 Overview of steroidogenic enzymes

Two major functional classes of enzymes are involved in the biosynthesis of all steroid hormones, namely the cytochrome P450 (CYP) and hydroxysteroid dehydrogenase (HSD) enzymes. The heme-containing CYP enzymes activate molecular oxygen utilizing NADPH as an electron donor. During catalysis, they incorporate one oxygen atom into the substrate while the other oxygen atom is reduced to water. This catalytic potential allows CYPs to catalyze a wide range of reactions, with hydroxylation and C—C bond cleavage being relevant reactions in steroidogenesis [9,10]. CYP enzymes involved in steroidogenesis can be divided into two groups based on their intracellular location and mode of electron transfer. CYP type I enzymes are located within the inner mitochondrial membrane and are dependent on ferredoxin and ferredoxin reductase for the delivery of their electrons from NADPH. Ferredoxin reductase is a flavoprotein that oxidizes NADPH and transfers electrons to ferredoxin, a small iron-sulfur protein, which acts as a mobile electron carrier, delivering the electrons to the CYP. The adrenally located ferredoxin reductase and ferredoxin are often also referred to as adrenodoxin reductase (AdxR) and adrenodoxin (Adx), respectively. CYP type II enzymes are found in the ER and are dependent on the electron donor enzyme cytochrome P450 oxidoreductase (POR) for electron delivery. POR contains a flavin adenine dinucleotide (FAD) and a flavin mononucleotide (FMN) allowing the enzyme to oxidize NADPH and reduce the CYP enzyme in a stepwise manner. The availability of NADPH is a vital aspect of CYP-catalyzed reactions, with redox partner ratios differentially influencing CYP activities [[11], [12], [13]].

The other main functional class of enzymes involved in steroidogenesis are the HSD enzymes which are dependent on NAD(P)H and NAD(P)^+^ co-factors. HSDs are subdivided into two distinct enzyme superfamilies based on their structural fold. These are the short chain dehydrogenases and aldo-keto reductases (AKR). The function of the HSD enzymes from both families is to catalyze the conversion of a given hydroxysteroid to its corresponding ketosteroid counterpart and vice versa, and in doing so, regulate the activity of the steroid at specific steroid receptors [14]. Most HSD-catalyzed reactions are mechanistically reversible and can function bi-directionally, although a prominent directionality is observed *in vivo* as a result of co-factor affinity and cellular redox status. An exception to this rule are the two HSD3B isoforms, HSD3B1 and HSD3B2, which catalyze an irreversible reaction, directly linked to the isomerization of the Δ^5^ double bond. These enzymes have dual catalytic activity and not only transform the hydroxy group on carbon 3 to a keto group but additionally isomerize the double bond from Δ^5^ to Δ^4^ [[15], [16], [17], [18]].

### 2.3 Overview of adrenal steroidogenesis

**Fig. 1:**
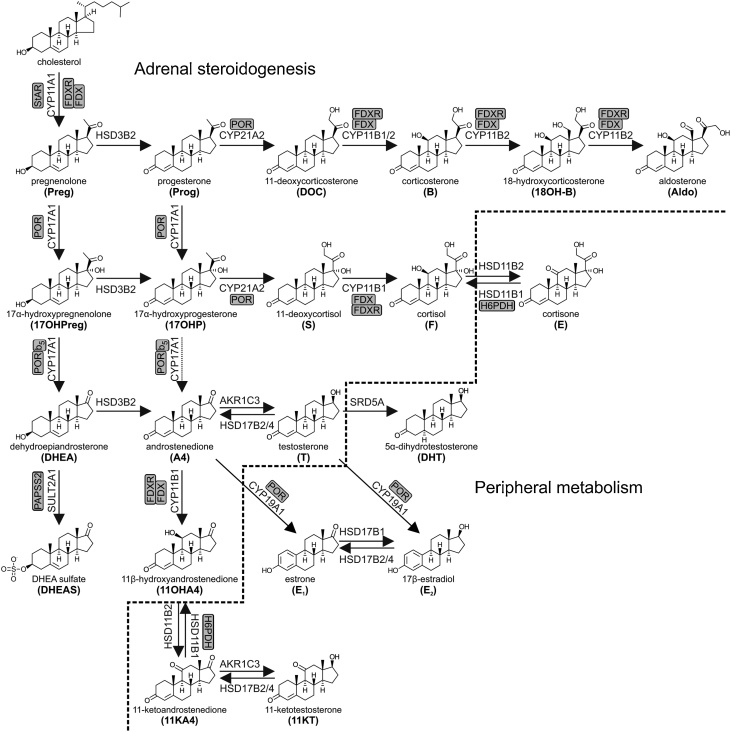
**Schematic overview of adrenal steroidogenesis and peripheral modulation of steroid bioactivity.** Arrows are labelled with the catalyzing enzyme and isoform where appropriate. Essential accessory proteins are also indicated: cytochrome *b*_5_ (b_5_); cytochrome P450 oxidoreductase (POR); ferredoxin (FDX); ferredoxin reductase (FDXR); hexose-6-phosphate dehydrogenase (H6PDH); PAPS synthase 2 (PAPSS2); steroidogenic acute regulatory protein (StAR).

The cortex of the adrenal gland is responsible for the biosynthesis of mineralocorticoids and glucocorticoids, as well as the production of adrenal androgen precursors and androgens, a function unique to higher primates [19,20]. The cortex is subdivided into three functional zones, each responsible for the production of a distinct steroid class due to the zone-specific expression of steroidogenic enzymes. The outer zone of the adrenal is termed the *zona glomerulosa* and expresses enzymes that catalyze the production of the major mineralocorticoid aldosterone under the control of the renin-angiotensin-aldosterone system. The middle zone, the *zona fasciculata*, is responsible for the production of the primary glucocorticoid, cortisol. Finally, the innermost zone, the *zona reticularis*, contributes to the formation of C_19_ androgen precursors including dehydroepiandrosterone (DHEA) and its sulfate (DHEAS), androstenedione (A4) and 11β-hydroxyandrostenedione (11OHA4) (Fig. 1). The hypothalamic-pituitary-adrenal (HPA) axis regulates the production of glucocorticoids and adrenal androgen precursors by the adrenal. In short, the hypothalamus produces corticotropin-releasing hormone (CRH) that stimulates corticotrope cells in the anterior pituitary to biosynthesize and release adrenocorticotropic hormone (ACTH) that in turn stimulates the adrenal gland to produce steroid hormones, specifically DHEA and cortisol [21,22]. Glucocorticoids complete the system by having a negative feedback effect on the pituitary, hypothalamus and the hippocampus, inhibiting further stimulation of the adrenal gland, while there is no feedback inhibition of the HPA axis by adrenal androgen precursors.

### 2.4 Overview of gonadal steroidogenesis

**Fig. 2:**
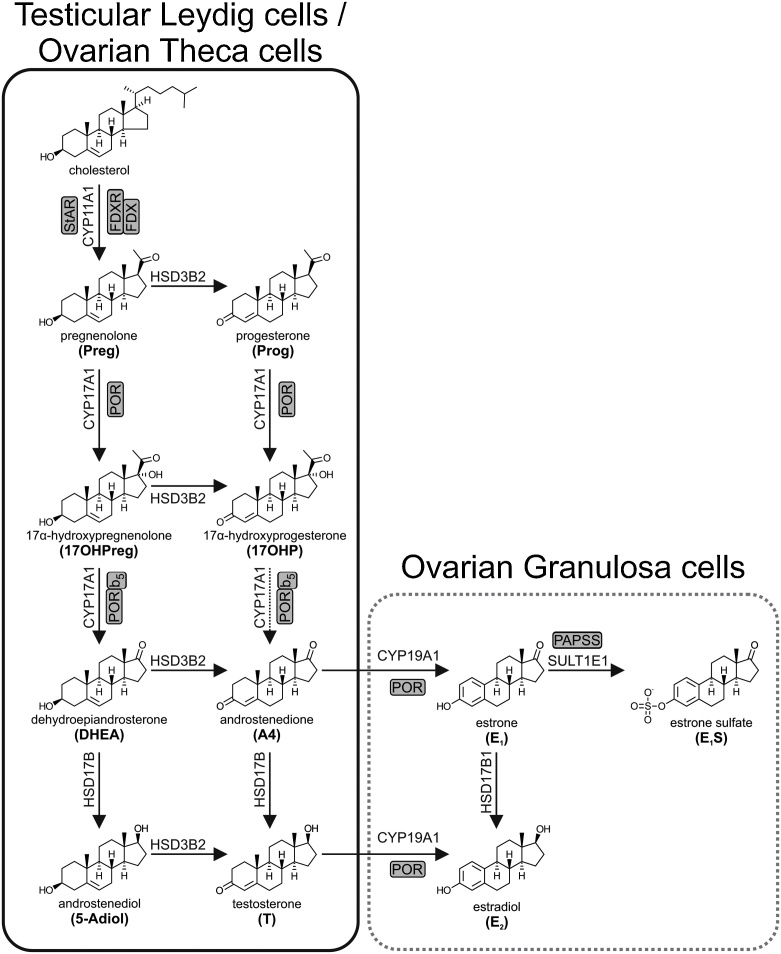
**Schematic overview of steroidogenesis in the gonads.** Steroidogenic pathways in the testicular Leydig cells are shown in the black box, while those in the ovaries are shown in the grey box and are further subdivided into the theca and granulosa cells. Arrows are labelled with the catalyzing enzyme and isoform where appropriate. Essential accessory proteins are also indicated: cytochrome *b*_5_ (b_5_); cytochrome P450 oxidoreductase (POR); ferredoxin (FDX); ferredoxin reductase (FDXR); PAPS synthase (PAPSS); steroidogenic acute regulatory protein (StAR).

Steroidogenesis in the gonads is tailored to the production of androgens and estrogens, with the corpus luteum additionally playing an important role in the production of the major endogenous progestogen, progesterone. Similar to the zonation of the adrenal, it is the cell-specific expression pattern of steroidogenic enzymes within each cell type that dictates steroid output (Fig. 2). Gonadal steroidogenesis is initiated by the development of the hypothalamic-pituitary-gonadal axis at puberty. The hypothalamus produces and secretes gonadotropin-releasing hormone (GnRH) in a pulsatile fashion, which in turn stimulates the production and secretion of luteinizing hormone (LH) from the pituitary. Androgens and estrogens provide negative feedback at the hypothalamus and pituitary level to suppress LH in men and women, respectively [23]. Gonadal steroidogenesis is also active during ‘minipuberty’, a short period of hypothalamic-pituitary-gonadal axis activation during the neonatal period [24].

### 2.5 Biosynthesis of specific steroid classes

#### 2.5.1 Mineralocorticoid production in the adrenal *zona glomerulosa*

Enzyme expression in the *zona glomerulosa* is tailored to produce the C_21_ mineralocorticoid, aldosterone. CYP11A1 converts cholesterol to pregnenolone, followed by the HSD3B2-catalyzed conversion of pregnenolone to the Δ^4^ steroid, progesterone. HSD3B2 is present in both the mitochondria and ER of *zona glomerulosa* cells [25,26]. Progesterone is subsequently converted to 11-deoxycorticosterone (DOC) by CYP21A2, which is abundantly expressed in the ER of the *zona glomerulosa* [26,27]. The lack of CYP17A1 expression in the *zona glomerulosa* [25] together with the abundant expression of HSD3B2 ensures that all steroid intermediates are directed towards aldosterone biosynthesis. Two isoforms of CYP11B are expressed in the *zona glomerulosa*, both with the ability to catalyze the 11β-hydroxylation of DOC yielding corticosterone. CYP11B2, which is also known as aldosterone synthase, additionally exhibits 18-hydroxylase and 18-methyl oxidase activity, which are required to convert corticosterone to aldosterone via the 18-hydroxycorticosterone intermediate [26,28,29].

#### 2.5.2 Glucocorticoid production in the adrenal *zona fasciculata*

The adrenal *zona fasciculata* is the site of glucocorticoid production. Pregnenolone, produced from the CYP11A1 catalyzed side-chain cleavage of cholesterol, is converted to 17α-hydroxyprogesterone (17OHP), the universal precursor of cortisol production, by HSD3B2 and CYP17A1 17α-hydroxylase activity. CYP21A2 subsequently catalyzes the conversion of 17OHP to 11-deoxycortisol, an obligatory step in the production of glucocorticoids. Finally, CYP11B1, located in the mitochondria of the *zona fasciculata* cells, facilitates the final step in glucocorticoid biosynthesis by catalyzing the conversion of 11-deoxycortisol to cortisol.

#### 2.5.3 Androgen biosynthesis

##### 2.5.3.1 The classic androgen biosynthesis pathway in the adrenal *zona reticularis* and the gonads

**Fig. 3:**
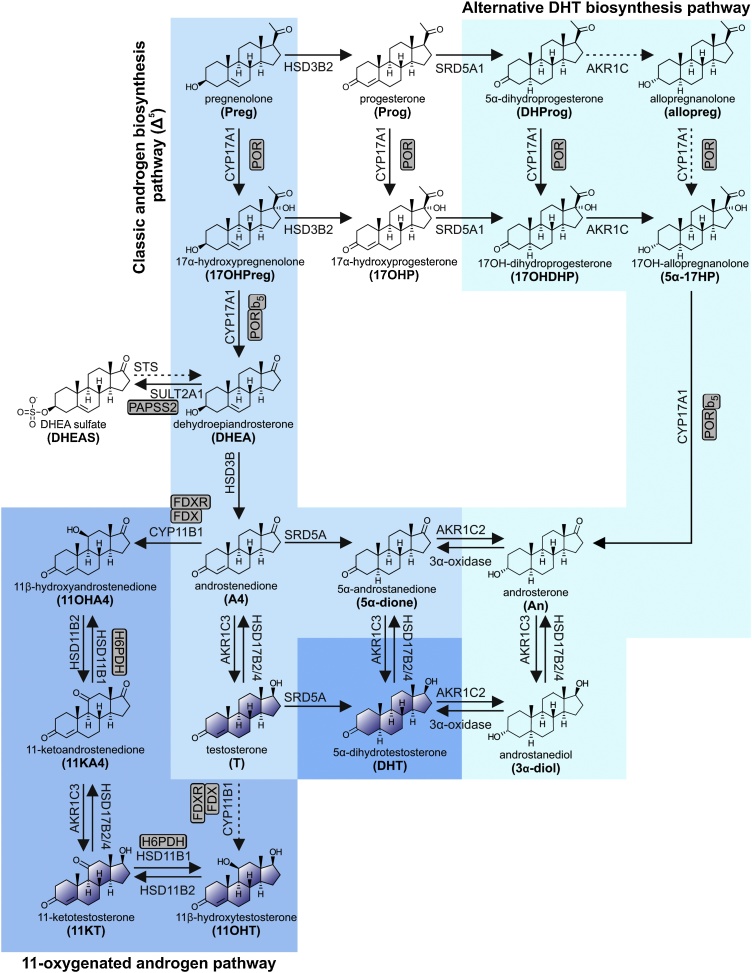
**Schematic overview of androgen biosynthesis**. Bioactive androgens (testosterone (T), 5α-dihydrotestosterone (DHT), 11-ketotestosterone (11KT) and 11β-hydroxytestosterone (11OHT) can be generated by three partially independent pathways which operate across multiple tissues: (1) the **classic Δ^5^ pathway**, (2) the **alternative DHT biosynthesis pathway**, and (3) the **11-oxygenated androgen pathway**. Arrows are labelled with the catalyzing enzyme and isoform where appropriate. Essential accessory proteins are indicated: cytochrome b5 (b_5_); cytochrome P450 oxidoreductase (POR); ferredoxin (FDX); ferredoxin reductase (FDXR); hexose-6-phosphate dehydrogenase (H6PDH); PAPS synthase 2 (PAPSS2); steroidogenic acute regulatory protein (StAR).

Androgen precursors and active androgens are produced by both the adult adrenal and gonads by the Δ^5^ pathway from pregnenolone to DHEA (Fig. 3). This pathway is also referred to as the ‘classic androgen biosynthesis pathway’. The CYP17A1-catalyzed 17α-hydroxylation of pregnenolone yields 17α-hydroxypregnenolone, which serves as the preferred substrate for the 17,20-lyase activity of CYP17A1, producing C_19_ steroids from C_21_ precursors [[30], [31], [32]]. It should be noted that the 17,20-lyase activity of CYP17A1 is dependent on augmentation by cytochrome b_5_ (CYB5A) in addition to electron transfer from POR [33].

The Δ^5^ pathway is active in the *zona reticularis* of the adrenal cortex, which only develops during adrenarche between the ages of 6–10, a process unique to humans and higher primates. During that time, the development of a distinct *zona reticularis* is accompanied by an extreme increase in the adrenal androgen precursor production due to the decreased expression of HSD3B2 in conjunction with increased CYB5A expression [[34], [35], [36], [37], [38]]. Some of the resulting DHEA is converted to androstenediol by AKR1C3, which exhibits minor expression in the *zona reticularis* [39]. However, the majority of DHEA is efficiently sulfated by the major DHEA sulfotransferase (SULT2A1), which is abundantly expressed in the *zona reticularis* [40]. This results in significant DHEAS output and accounts for DHEAS being the most abundant steroid in circulation (Table 2) [39,41]. Other Δ^5^ steroids, e.g. pregnenolone, 17α-hydroxypregnenolone and androstenediol, can also be released in their respective sulfated form [[42], [43], [44]]. Moreover, DHEA and androstenediol can all be converted to their corresponding Δ^4^ products by the low levels of HSD3B2, yielding A4 and testosterone, respectively. A4 can serve as an additional substrate for AKR1C3, yielding testosterone [45]. An age-related gradual decrease in adrenal androgen secretion and excretion, known as adrenopause, occurs starting in the fourth decade of life and is associated with a decrease in the *zona reticularis* cell layer and cell function. This reaches a minimum by the age of 70, with only about 5–10% of the peak levels observed in young adulthood [46].

Like in the *zona reticularis*, the Leydig cells of the testes follow the Δ^5^ pathway due to the co-expression of CYP17A1 and CYB5A (Fig. 2). However, in the Leydig cells subsequent metabolism is directed at testosterone biosynthesis due to the absence of SULT2A1 and the expression of HSD3B2 and HSD17B3. DHEA is then converted to testosterone via A4 or androstenediol through the action of HSD3B2 and HSD17B3, respectively, with HSD17B3 playing a key role in testicular androgen biosynthesis [47,48]. AKR1C3 expression has been reported in Leydig cells and may also contribute to testosterone production [49,50]. Testicular steroid output is predominantly testosterone, with lower levels of A4 and DHEA also being released into circulation [[51], [52], [53]]. Androgen and androgen precursor production by the ovary follows a similar route to that of the testes (Fig. 2).

##### 2.5.3.2 Peripheral tissue activation of androgen precursors

It should be highlighted that with the exception of testosterone produced by the testes, the vast majority of C_19_ steroids produced by the adrenal and ovaries are inactive androgen precursors. These can, however, subsequently be converted to active androgens in target cells of androgen action that express the required enzymatic machinery (Fig. 3) [45]. More specifically, DHEA can be converted to A4 by peripheral HSD3B1, with A4 serving as the substrate for the production of testosterone by AKR1C3 [54]. Subsequent 5α-reduction of testosterone yields the more potent androgen 5α-dihydrotestosterone (DHT), with this step therefore serving as a target-specific amplification of the androgen signal, a pre-receptor activation of testosterone to DHT [45]. A4 can also be 5α-reduced to 5α-androstanedione prior to conversion to DHT by AKR1C3, thereby bypassing testosterone (Fig. 3) [45,55]. Indeed, the enzyme steroid 5α-reductase 1 (SRD5A1) catalyzes the 5α-reduction of A4 more efficiently than that of testosterone [56,57]. This so called “alternate 5α-androstanedione” pathway is favored in tissues with predominant SRD5A1 expression and in conditions such as castration-resistant prostate cancer in which the expression of *SRD5A1* is upregulated and that of *SRD5A2* is downregulated [55,58]. As SRD5A2 does not demonstrate the same substrate preference, tissues expressing this isoform are thought to follow the more conventional pathway of conversion to testosterone, prior to 5α-reduction [56]. Moreover, although the liver undoubtedly makes the major contribution to steroid metabolism, most peripheral tissues also possess enzymatic machinery for inactivation of androgens (both those obtained from circulation and those produced from inactive precursors) by phase 1 and 2 metabolism (Section 3). This process of peripheral cell specific activation and inactivation is known as pre- and post-receptor steroid metabolism or steroid ” intracrinology” [45,[59], [60], [61]].

##### 2.5.3.3 The alternative DHT biosynthesis pathway

In selected circumstances, such as CYP21A2 deficiency and during fetal development (Section 2.6), accumulation of progesterone and 17OHP in circulation can lead to the activation of an alternative pathway of DHT biosynthesis (Fig. 3). This is sometimes referred to as the “backdoor pathway” to DHT [62]. To enter this pathway, progesterone and 17OHP are 5α-reduced by SRD5A1 to yield 5α-dihydroprogesterone and 17α-hydroxydihydroprogesterone, respectively. These are then subsequently converted to allopregnanolone and 17α-hydroxyallopregnanolone by the 3α-reductase activity of AKR1C enzymes. Allopregnanolone can then be converted to 17α-hydroxyallopregnanolone, which serves as an excellent substrate for the 17,20-lyase activity of CYP17A1 [30], yielding androsterone. Androsterone, considered an inactive metabolite of DHT under normal circumstances, can then be reactivated by the sequential 17β-reduction and 3α-oxidase reactions [[63], [64], [65]]. Androsterone has been shown to be the principle circulating androgen precursor for the alternative DHT biosynthesis pathway in the male fetus during the second trimester. Interestingly, placental progesterone has been suggested to serve as substrate for androsterone biosynthesis in the male fetus via the alternative pathway which occurs across several non-gonadal fetal tissues [66].

##### 2.5.3.4 The 11-oxygenated androgen biosynthesis pathway

Within the adrenal, A4 can serve as a substrate for CYP11B1, yielding the 11-oxygenated androgen precursor, 11OHA4 [67], with conversion of testosterone to 11β-hydroxytestosterone (11OHT), also occurring (Fig. 3). However, 11OHA4 is by far the predominant product due to the significantly higher levels of A4 produced in the *zona reticularis* [39]. Notably, the addition of exogenous testosterone does not lead to increased 11-oxygenated androgen output, thereby confirming that only locally produced substrates (primarily A4) can be 11β-hydroxylated [68]. Low levels of 11-ketoandrostenedione (11KA4) and 11-ketotestosterone (11KT) have also been reported in adrenal vein samples and are suggested to result from some HSD11B2 activity in the adrenal [39]. However, differences in the concentration of 11KA4 measured in the adrenal vein and inferior vena cava suggest that 11KA4 is predominantly produced from conversion of 11OHA4 in peripheral tissue expressing HSD11B2 such as the kidney [39,69]. 11KA4 in turn serves as a substrate for AKR1C3 expressed in peripheral tissues such as adipose tissue, yielding 11KT, which binds and activates the human androgen receptor with an affinity and potency similar to that of testosterone [45,[70], [71], [72]]. Indeed, a recent study has shown that AKR1C3 catalyzes the conversion of 11KA4 to 11KT with an 8-fold higher efficiency than that of A4 to testosterone, which may account for higher levels of peripheral activation [73]. Peripheral or intracrine activation of 11-oxygenated androgens may therefore play a vital role in regulating their physiological activity. Interestingly, activation/inactivation of glucocorticoids and 11-oxygenated androgens work in an antiparallel manner, with the 11β-hydroxy derivative being the active glucocorticoid [74,75], while the 11-keto androgens are more potent than their 11β-hydroxy counterparts [76].

#### 2.5.4 Progestogen and estrogen biosynthesis by the ovary

Steroidogenesis within the ovary is compartmentalized in a cell-specific manner, with the theca cells mainly producing A4 and progesterone, while the granulosa cells complete the biosynthesis of 17β-estradiol (Fig. 2). Ovarian steroidogenesis originates in the theca cells with the production of A4 via the Δ^5^ pathway. The resulting A4 (and testosterone) enters the granulosa cells where the expression of CYP19A1 (aromatase) results in estrogen biosynthesis. Ovarian steroid output varies considerably during the course of the menstrual cycle – 17β-estradiol is the primary steroid produced during the follicular phase, while progesterone is the principal steroid during the luteal phase [[77], [78], [79]].

In addition to *de novo* steroidogenesis, it is important to note that the ovary also utilizes circulating DHEA and A4 of adrenal origin for the biosynthesis of androgens and estrogens [80]. Suppression of adrenal androgen output by dexamethasone in healthy, young women with a regular menstrual cycle leads to a 90% decrease in DHEA(S) output, but also reduces circulating testosterone and DHT concentrations to one third of their respective baseline concentrations [81]. While A4 can be metabolized to testosterone by AKR1C3 in the theca cells, the majority diffuses to the granulosa cells where the high expression levels of CYP19A1 results in the production of estrone. HSD17B1 subsequently catalyzes the conversion of estrone to 17β-estradiol, under the regulation of FSH. Testosterone diffusing from the theca cells also serves as the substrate for CYP19A1, directly contributing to 17β-estradiol production. Theca cells also express high levels of the estrogen sulfotransferase enzyme SULT1E1, which preferentially sulfates estrone yielding the relatively abundant estrone sulfate measured in circulation [[82], [83], [84]]. However, quantitatively estrogens circulate at significantly lower levels than androgens. Indeed, androgen secretion by the theca cells surpasses the secretion of estrogens, while progesterone is the primary progestogen produced [85]. It should also be noted that the peripheral aromatization of C_19_ steroids plays an important role in peripheral estrogen production, particularly following menopause as outlined in Section 4.4 below.

## 3 Principles of steroid metabolism and excretion

Steroids are inherently lipophilic molecules. Metabolic conversions are therefore required to increase their water-solubility and enable efficient excretion in urine and bile (Table 1). This metabolism is traditionally divided into two sequential stages, namely phase 1 and phase 2 reactions [86]. Phase 1 reactions alter the biological activity and at the same time add or reveal functional groups that function as targets for subsequent phase 2 reactions. Phase 2 reactions are conjugation reactions that ultimately inactivate the compound and increase polarity and water solubility, thereby facilitating urinary and biliary excretion. Additionally, conjugation with a charged group limits transport over membranes to active transport, thereby allowing for the concentration of the metabolite on one side [87,88]. The major phase 1 reactions for steroids are the reduction of the 3-keto-Δ^4^ motif, the interconversion of hydroxy- and keto-groups by HSDs/oxoreductases and additional hydroxylations by CYPs.

**Table 1:** List of common circulating steroids and their major urine metabolites. Common abbreviations are shown in brackets.

Serum steroid and abbreviation	Major urine metabolite and abbreviation (unconjugated form)
**General precursors**	
pregnenolone, 5-pregnen-3β-ol-20-one **(PREG; P5)**	5-pregnenediol, 5-pregnen-3β,20α-diol **(5PD)**
progesterone, 4-pregnen-3,20-dione **(PROG; P4)**	pregnanediol, 5β-pregnan-3α,20α-diol **(PD)**
17α-hydroxypregnenolone, 5-pregnen-3β,17α-diol-20-one **(17Preg; 17OHPreg; 17P5)**	5-pregnenetriol, 5-pregnen-3β,17α,20α-triol **(5 P T)**
17α-hydroxyprogesterone, 4-pregnen-17α-ol-3,20-dione **(17OHP; 17OHProg; 17P4)**	pregnanetriol, 5β-pregnan-3α,17α,20α-triol **(PT)**
	17α-hydroxypregnanolone, 5β-pregnan-3α,17α-diol-20-one **(17HP)**

**Mineralocorticoids and their precursors**	
11-deoxycorticosterone, 4-pregnen-21-ol-3,20-dione **(DOC)**	tetrahydro-11-deoxycorticosterone, 5β-pregnan-3α,21-diol-20-one **(THDOC)**
corticosterone, 4-pregnene-11β,21-diol-3,20-dione **(CORT; B)**	tetrahydro-11-dehydrocorticosterone, 5β-pregnan-3α,21-diol-11,20-dione (**THA**)
	5α-tetrahydro-11-dehydrocorticosterone, 5α-pregnan-3α,21-diol-11,20-dione (**5α-THA**)
	5β-tetrahydrocorticosterone, 5β-pregnan-3α,11β,21-triol-20-one (**THB**)
	5α-tetrahydrocorticosterone, 5α-pregnan-3α,11β,21-triol-20-one (**5α-THB**)
18-hydroxycorticosterone, 4-pregnene-11β,18,21-triol-3,20-dione **(18OHCORT; 18OHB; 18B)**	18-hydroxytetrahydro-11-dehydrocorticosterone, 5β-pregnan-3α,18,21-triol-11, 20-dione (**18OHTHA**)
aldosterone, 4-pregnene-11β,21-diol-3,20-dione-18-al **(ALDO)**	tetrahydroaldosterone, 5β-pregnan-3α,11β,21-triol-20-one-18-al **(THAldo)**

**Glucocorticoids and their precursors**	
11-deoxycortisol, 4-pregnen-17α,21-diol-3,20-dione **(S)**	tetrahydro-11-deoxycortisol, 5β-pregnan-3α,17α,21-triol-20-one **(THS)**
21-deoxycortisol, 4-pregnene-11β,17α-diol-320-dione	pregnanetriolone, 5β-pregnan-3α,17α,20α-triol-11-one **(PTONE)**
cortisol, 4-pregnene-11β,17α,21-triol-320-dione **(F)**	6β-hydroxycortisol, 4-pregnen-6β,11β,17α,21-tetrol-3,20-dione **(6β-OHF)**
	cortisol, 4-pregnene-11β,17α,21-triol-3,20-dione **(F)**
	tetrahydrocortisol, 5β-pregnan-3α,11β,17α,21-tetrol-20-one **(THF)** 5α-tetrahydrocortisol, 5α-pregnan-3α,11β,17α,21-tetrol-20-one **(5α-THF)** **α-cortol**, 5β-pregnan-3α,11β,17α,20α,21-pentol **β-cortol**, 5β-pregnan-3α,11β,17α,20β,21-pentol 11β-hydroxyeticholanolone, 5β-androstan-3α,11β-ol-17-one **(11β-OHEt)**
cortisone, 4-pregnene-17α,21-diol-3,11,20-trione **(E)**	cortisone **(E)**
	tetrahydrocortisone **(THE)**
	**α-cortolone**, 5β-pregnan-3α,17α,20α,21-tetrol-11-one
	**β-cortolone**, 5β-pregnan-3α,17α,20β,21-tetrol-11-one
	11-ketoetiocholanolone, 5β-androstan-3α-ol-17,11-dione (**11ketoEt**)

**“Hybrid steroids”**	
18-hydroxycortisol, 4-pregnene-11β,17α,18,21-tetrol-3,20-dione **(18OHF)**	18-hydroxycortisol, 4-pregnene-11β,17α,18,21-tetrol-3,20-dione **(18OHF)**
18-oxo-cortisol, 4-pregnene-11β,17α,21-triol-3,20-dione-18-al **(18oxoF)**	18-oxo-tetrahydrocortisol, 4-pregnene-11β,17α,21-triol-3,20-dione-18-al **(18oxoTHF)**

**Androgen precursor metabolites**	
dehydroepiandrosterone sulfate, 5-androsten-3β-sulfate-17-one **(DHEAS)**	dehydroepiandrosterone, 5-androsten-3β-ol-17-one **(DHEA)**
dehydroepiandrosterone, 5-androsten-3β-ol-17-one **(DHEA)**	dehydroepiandrosterone, 5-androsten-3β-ol-17-one **(DHEA)**
	16α-hydroxydehydroepiandrosterone **(16α-DHEA)**
androstenedione, 4-androsten-3,17-dione **(A4)**	androsterone, 5α-androstan-3α-ol-17-one **(An; AST)**
	etiocholanolone, 5β-androstan-3α-ol-17-one **(Et)**
11β-hydroxyandrostenedione, 4-androsten-11β-ol-3,17-dione **(11OHA4; 11β-OHA4)**	11β-hydroxyandrosterone, 5α-androstan-3α,11β-diol-17-one **(11β-OHAn; 11βOHAST))**
17-hydroxyallopregnanolone, 5α-pregnane-3α,17α-diol-20-one (**5α-17HP**)	17-hydroxyallopregnanolone, 5α-pregnan-3α,17α-diol-20-one (**5α-17HP**)

**Androgen metabolites**	
testosterone, 4-androsten-17β-ol-3-one **(T)**	androsterone, 5α-androstan-3α-ol-17-one **(An; AST)**
	etiocholanolone, 5β-androstan-3α-ol-17-one **(Et)**
5α-dihydrotestosterone, 5α-androstan-17β-ol-3-one **(DHT; 5α-DHT)**	androsterone, 5α-androstan-3α-ol-17-one **(An; AST)**

**Table 2:** Graphical representation of the circulating serum steroid metabolome. Major circulating steroids are shown divided into six concentration ranges illustrating their relative contribution to the total circulating steroid pool.

					
1-10 μmol/L	100-1000 nmol/L	10-100 nmol/L	1-10 nmol/L	100-1000 pmol/L	<100 pmol/L
**DHEAS**	**cortisol**	**corticosterone**	**17OHP**	**17OHP**	**17β-estradiol**
		**cortisone**	(male)	(female, follicular phase)	(male)
		**DHEA**	**17OHP**	**DOC**	**17β-estradiol**
		**testosterone**	(female, luteal phase)	**18OH-corticosterone**	(female, postmenopausal)
		(male)	**pregnenolone**	**aldosterone**	**estrone**
		**progesterone**	**17OH-pregnenolone**	**18-oxocortisol**	(male)
		(female, luteal phase)	**11-deoxycortisol**	**11OHT**	**estrone**
			**18-hydroxycortisol**	**progesterone**	(female, postmenopausal)
			****A4****	**(male)**	
			**11OHA4**	**progesterone**	
			**11KA4**	(female, postmenopausal)	
			**testosterone**	**17β-estradiol**	
			(female)	(female, premenopausal)	
			**11KT**	**estrone**	
			**progesterone** (female, follicular phase)	(female, premenopausal)	

Although the liver undoubtedly makes the major contribution to steroid metabolism, most peripheral tissues also possess enzymatic machinery for aspects of both steroid activation and subsequent inactivation by phase 1 and 2 metabolism. This localized enzyme expression controls the local steroid milieu by precursor activation and inactivation according to tissue-specific needs, a mechanism known as intracellular pre-and post-receptor metabolism or “intracrinology” [59].

While phase 1 and phase 2 reactions are classically believed to be sequential, more recent studies have shown the metabolism of conjugated steroids by phase 1 enzymes [89]. Additionally, some steroids can directly undergo phase 2 metabolism without being subjected to a phase 1 reaction, e.g. testosterone can be directly conjugated at its 17β-hydroxy group and corticosteroids through 21-sulfation [90]. Despite these and other shortcomings [91], the traditional classification in phase 1 and 2 reactions remains helpful to structure the wide range of reactions and this classification is used below to guide the reader through the metabolism of endogenous steroids.

### 3.1 Phase 1 metabolism of steroids

#### 3.1.1 Steroid A-ring reduction

**Fig. 4:**
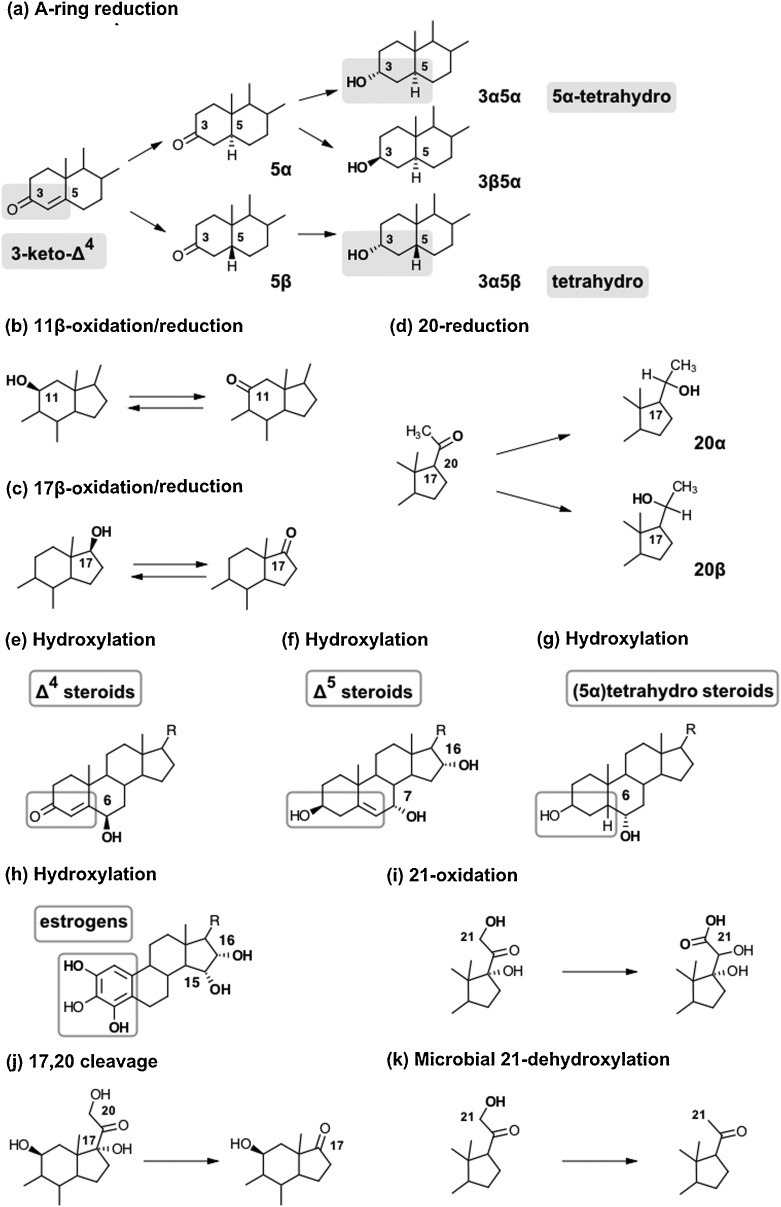
**Schematic overview of the major phase 1 reactions contributing to steroid metabolism**. **(a) A-ring reduction** to (5α)tetrahydro metabolites. The formation of 3β,5β-tetrahydro metabolites is sterically unfavorable (not shown). **(b) 11β-oxidation/reduction** by HSD11B1 modulates the bioactivity of glucocorticoids, mineralocorticoids and 11-oxygenated androgens. **(c) 17β-oxidation/reduction** regulates the bioactivity of androgens and estrogens. **(d) 20-reduction** to a hydroxy group with α- or β-stereochemistry. **(e–h) Hydroxylations**: major positions are indicated for different structural steroid classes. **(i) 21-oxidation** leading to the formation of the so-called cortolic acids from cortisol. *(j)***17,20-cleavage**: cortisol, cortisone and their metabolites can undergo metabolism by 17,20-lyase activity. **(k) Microbial 21-dehydroxylation**: steroids excreted with bile can undergo metabolism by the gut microbiome prior to reabsorption.

Reduction of the steroid A-ring 3-keto-Δ^4^ motif is an essential step for the inactivation of gluco- and mineralocorticoids and controls the peripheral activation and inactivation of androgens. A-ring reduction consists of two sequential reductions, namely the reduction of the Δ^4^-double bond followed by the reduction of the 3-keto group to a hydroxy group [92] (Fig. 4(a)). This leads to the production of a 3α/β-hydroxy-5α/βH motif common to the biologically inactive, excreted metabolites. Among these, the 5β/3α-metabolites are referred to as “tetrahydro”, while 5α/3α-metabolites are referred to as “5α-tetrahydro”. Importantly, 5α/β-reduction is irreversible, with the stereochemistry of this reduction playing an important role in regulating the biological activity of androgens (Section 4.3.2) [93].

5α-Reduction is catalyzed by steroid 5α-reductase (SRD5A) enzymes of which there are three main isozymes. However, only two of these, SRD5A1 and SRD5A2, function as genuine steroid 5α-reductases. SRD5A1 is mainly expressed in the liver and peripheral tissues [94], while SRD5A2 is expressed mainly in male reproductive and genital tissues, with its disruption leading to disordered sex development in 46,XY individuals [95]. SRD5A3 appears to have only minor steroid 5α-reductase activity, but has been shown to play an important role in N-linked protein glycosylation [[96], [97], [98]]. In addition, two partially homologous SRD5A genes have been identified (SRD5A2L2 and GPSN2), but have been shown to be involved in the elongation of very long chain fatty acids [99]. Steroid 5β-reduction is catalyzed by the aldo-keto-reductase (AKR) family member AKR1D1, which is primarily expressed in the liver. It is the only human enzyme catalyzing the 5β-reduction of 3-keto-Δ^4^ steroids and bile acids [100]. AKR1D1 deficiency leads to severely reduced or abolished urinary 5β-reduced steroid excretion and hepatic failure [101].

Due to differential tissue expression patterns, with SRD5A isoforms being widely expressed in peripheral tissues including the liver and AKR1D1 expression being limited to the liver, 5α-reduced metabolites inform about global metabolism while 5β-reduced metabolites predominantly reflect hepatic reduction only. Moreover, SRD5As and AKR1D1 exhibit different catalytic efficiencies towards structurally different steroids [57,100], with the result that 5α- and 5β-reduced metabolites are produced with different ratios for different structural classes of steroids.

The second step of the A-ring reduction is the reduction of the 3-keto group to a hydroxy group. These reactions are catalyzed by members of the aldo-keto reductase family, namely AKR1C1, AKR1C2, AKR1C3 and AKR1C4. Of these, AKR1C4 is thought to be a liver-specific enzyme which works in concert with AKR1D1, yielding 5β,3α-metabolites. The other isozymes are expressed in different peripheral tissue in an tissue-specific manner [54].

While 5α-reduced steroids can be converted to both their 3α- or 3β-hydroxy epimers (with the 3α-reduction generally being more efficient), 5β-reduced steroids are predominantly converted to the 3α-hydroxy epimer as the 5β-reduced bent confirmation of the A/B-ring sterically does not allow binding in the AKR1C active site for 3β-reduction [102,103]. AKR1C2 is the major isoform for 3-reduction to the 3α-hydroxyepimer in peripheral tissue, while AKR1C1 is the most important isoform for the formation of the 3β-hydroxyepimer [102]. Moreover, AKR1C enzymes are multifunctional and also function as 20α- and 17β-HSDs, with different efficiencies, stereoselectivities and tissue specific expression [104]. For a comprehensive review of these enzymes see [54].

#### 3.1.2 Hydroxysteroid dehydrogenation and reduction

The interconversion of hydroxy- and keto-groups (Fig. 4(b–d)) at positions 11, 17 and 20, greatly contribute to the regulation of steroid activity via their receptors. These reactions are catalyzed by members of the short-chain dehydrogenase/reductase (SDR) superfamily and the AKR superfamily using NAD(P)^+^/H and are typically reversible. While most of these enzymes are bidirectional *in vitro*, *in vivo* directionality is dictated by co-factor affinity, cellular redox status and pH [54,[105], [106], [107]].

##### 3.1.2.1 11β-hydroxysteroid dehydrogenases

Two isoforms of HSD11B play a key role in regulating glucocorticoid inactivation and reactivation by catalyzing the interconversion of 11β-hydroxy- and 11-ketosteroids (Fig. 4(b)). Thereby, they modulate systemic and tissue-specific glucocorticoid action [74,108]. Additionally, they are involved in the regulation of mineralocorticoid and 11-oxygenated androgen activity.

HSD11B1 is a bidirectional enzyme, but primarily catalyzes the reduction of 11-ketosteroids *in vivo* as colocalized hexose-6-phosphate dehydrogenase (H6PDH) regenerates NADPH required for its cortisone reductase activity, mainly activating cortisone to cortisol [109]. Conversely, HSD11B2 functions exclusively as an oxidative enzyme, inactivating cortisol to cortisone [108]. Both isoforms are involved in the metabolism of glucocorticoids and 11-oxygenated androgens as described in Sections 4.2.1 and 4.3.2. Moreover, both HSD11B isoforms also act on 7-oxygenated C_19_ steroids whereby HSD11B1 functions as an epimerase interconverting the 7α- and 7β-hydroxy-stereoisomers via a 7-keto-intermediate, while HSD11B2 only oxidizes the 7α-hydroxy-stereoisomer [110,111].

##### 3.1.2.2 17β-hydroxysteroid dehydrogenases

Enzymes from the SDR and AKR superfamilies regulate the activity of androgens and estrogens by catalyzing the interconversion of bioactive 17β-hydroxy- and inactive keto- containing forms (Fig. 4(c)). Excreted metabolites are therefore predominantly in the keto-form [112]. To date, 14 human enzymes with 17β-HSD/oxoreductase activities have been identified [105,106,113]. Generally, these enzymes are multi-functional and often have overlapping substrate specificities and expression patterns, allowing for redundant enzymes to cover in case of deficiency of another enzyme [107,114]. However, certain enzymes have been identified as major catalysts for specific reactions in androgen and estrogen metabolism as described in Sections 4.3.2 and 4.4.1.

##### 3.1.2.3 20-reduction

Glucocorticoids and progesterone can be modified by 20-reduction with α- or β-stereochemistry prior to excretion (Fig. 4(d)). Of note, the direct 20α-reduction of progesterone terminates its progestogenic activity and is predominantly catalyzed by AKR1C1 [104,115]. 20-reduction of glucocorticoids is primarily observed for downstream tetrahydrometabolites as described in Section 4.2.2.

#### 3.1.3 Cytochrome P450-catalyzed steroid oxidations

In addition to the steroidogenic CYP enzymes described in Section 2.2, hepatic xenobiotic-metabolizing members of the CYP superfamily are able to modify steroid hormones and generate a plethora of minor steroid metabolites [116]. The reaction repertoire of these enzymes for steroids includes hydroxylation reactions, further oxidations, and C—C bond cleavages [9,10]. Hepatic CYPs are promiscuous enzymes accepting a wide range of substrates with low stereo- and regioselectivity compared to their steroidogenic counterparts. Therefore, several CYPs can contribute to the same reaction and the high variation of their expression levels can make it difficult to assess the enzyme(s) making the dominant contribution to a specific reaction, with at least 17 hepatic CYPs potentially participating in the metabolism of steroids [[116], [117], [118]].

A high inter- and intra-individual variability of hepatic CYP activity results from the strong potential for induction by pharmacological and natural compounds, the high frequency and number of polymorphisms, and promoter and copy number variants [119,120]. In addition, differential expression profiles of functionally different isoforms of the CYP3A subfamily during prenatal and early postnatal life complicate the assessment of hepatic steroid metabolism. CYP3A4 is the most abundant CYP expressed in the adult liver [121,122] and makes the major contribution to steroid metabolism. CYP3A5 catalyzes a comparable range of reactions as CYP3A4 but its role in drug and steroid metabolism is limited due to its generally low activity and expression in a relatively small percentage of individuals [[123], [124], [125]]. CYP3A7 is the major CYP3A isoform in prenatal and early postnatal life and differs from CYP3A4 in terms of expression and function. CYPs also contribute to steroid metabolism in several extra-hepatic tissues, including the brain, breast and prostate [[126], [127], [128], [129]].

##### 3.1.3.1 Steroid hydroxylations

The hydroxylation by hepatic CYPs inactivates the steroids and increases their polarity and water solubility. In some cases, the additional hydroxy groups also serve as sites for conjugation by phase 2 metabolism. CYP3A4-catalyzed 6β-hydroxylation is the most common hydroxylation for Δ^4^ steroids, e.g. cortisol (Fig. 4(e)), while 6α-hydroxylated pregnanolones are quantitatively important urine steroid metabolites during pregnancy. Tetrahydro and hexahydro C_21_ steroids (e.g. THE and the cortolones) hydroxylated at 1β- and 6α- are quantitatively important during the perinatal period [130]. The enzymes responsible for these hydroxylations is uncertain. The differential substrate specificity, regioselectivity and catalytic activity of CYP3A4 and CYP3A7 and the dynamic expression pattern of the two isoforms throughout fetal development and the first year of life lead to substantial changes in the hepatic steroid metabolome during this period of life. CYP3A7 is highly expressed in fetal liver and up to 6 months postnatal but expression levels gradually decrease over this time. CYP3A4 levels are low in the fetus and newborn compared to the adult. Thus, there is a switch from CYP3A7 to CYP3A4 during the first months after birth. Additionally, the total liver CYP3A content is significantly higher prenatally followed by a reduction after birth reaching plateau at 6 months [131,132].

In terms of Δ^5^ steroids, e.g. DHEA, 16α-hydroxylation is the most frequent hydroxylation detected in adults followed by 7α-hydroxylation (Fig. 4(f)), while 16β-, 21-, 18- and 15β-hydroxylation are also observed in neonates [[133], [134], [135], [136]].

Interestingly, hepatic CYPs can also perform some reactions that are classically catalyzed by steroidogenic CYPs (11β-, 17α- and 21-hydroxylation) [[137], [138], [139]]. In fact, CYP2C19 and CYP3A4 can 21-hydroxylate progesterone and pregnenolone, possibly partially compensating mineralocorticoid deficiency in CAH due to 21-hydroxylase deficiency; however, the two enzymes are not capable of catalyzing the 21-hydroxylation of 17OHP to 11-deoxycortisol [140].

Estrogens can be hydroxylated in various different positions by a number of CYPs [141]. The formation of catecholestrogens by 2- or 4-hydroxyation are the dominant reactions (Fig. 4(h)). However, during pregnancy, estriol, which has a 16α-hydroxygroup, is the main metabolite of fetal DHEA. Estriol predominately originates from the aromatization of 16α-hydroxy C_19_ steroids by the placenta (Section 2.6.2).

##### 3.1.3.2 Additional steroid oxidations

CYPs can further oxidize hydroxy groups to their respective keto, aldehyde and carboxylic acids. For example, 6-keto metabolites can be produced from their hydroxy precursors [141]. 21-carboxylic acid formation from 21-hydroxysteroids is also possible (Fig. 4(i)) [[142], [143], [144], [145], [146]].

##### 3.1.3.3 Could a empty line be added before these subheadings as the heading is difficult to distinguish in the pdf C—C bond cleavages

CYPs can also catalyze oxidative C—C bond cleavages in multi-step reactions [10]. Examples for such reactions from steroid biosynthesis are the side-chain cleavage of cholesterol catalyzed by CYP11A1 and the 17,20-lyase activity of CYP17A1 producing C_19_ steroids from C_21_ substrates (Section 2.5.3) [113]. Hepatic CYPs may employ similar mechanisms to catalyze the 17–20 cleavage of 17α,21-dihydroxypregnanes (Fig. 4(j)).

##### 3.1.3.4 Could a empty line be added before these subheadings as the heading is difficult to distinguish in the pdf Contributions of the gut microbiome

Metabolism by the gut microbiome is relevant for C_17_-deoxy corticosteroids, e.g. mineralocorticoids and their precursors, which have a high biliary excretion [147]. The resulting metabolites can be reabsorbed into the portal system and undergo further metabolism in the liver and kidney before being excreted with the urine. Reactions catalyzed by different strains of gut bacteria include (**1**) A-ring reduction, (**2**) reduction of the Δ^5^-double bond, (**3**) reduction of 17-keto estrogens and 17-keto androstenes, (**4**) 17,20-cleavage of 17α-hydroxysteroids, and (**5**) 16α- and 21-dehydroxylation (Fig. 4(k)) [[148], [149], [150], [151], [152], [153], [154], [155], [156]]. Additionally, reductive 20α/β-HSDs are active in gut bacteria [151]. Unsurprisingly, steroid metabolism by gut bacteria has been shown to be influenced by the administration of antibiotics [157].

### 3.2 Phase 2 metabolism of steroids

The classic phase 2 conjugation reactions – sulfation and glucuronidation – increase the polarity and water solubility of the steroids and thereby facilitate their excretion and concentration in the urine. Mechanistically, these conjugation reactions proceed via two subsequent, enzymatically catalyzed reactions: (**1**) the activation of the moiety to be attached and (**2**) the transfer of the moiety from the activated donor onto a hydroxy group of the steroid. While the conjugated product is generally considered to be biologically inactive, rare exceptions have been identified [91]. Importantly, steroid sulfation is reversible and sulfated steroids can be hydrolyzed to free steroids by steroid sulfatase (STS), while glucuronidation is irreversible in humans, with the exception of the activity of some gut bacteria (Section 3.1.3.4). Notably, bis-conjugation with the same (e.g. bis-sulfation) [[158], [159], [160], [161], [162]] or two different groups (e.g. sulfate and glucuronic acid) are possible [163]. Other conjugation reactions include the methylation of catechol estrogens, conjugation with cysteine or glutathione, and esterification with fatty acids as outlined below.

#### 3.2.1 Steroid sulfation and desulfation

**Fig. 5:**
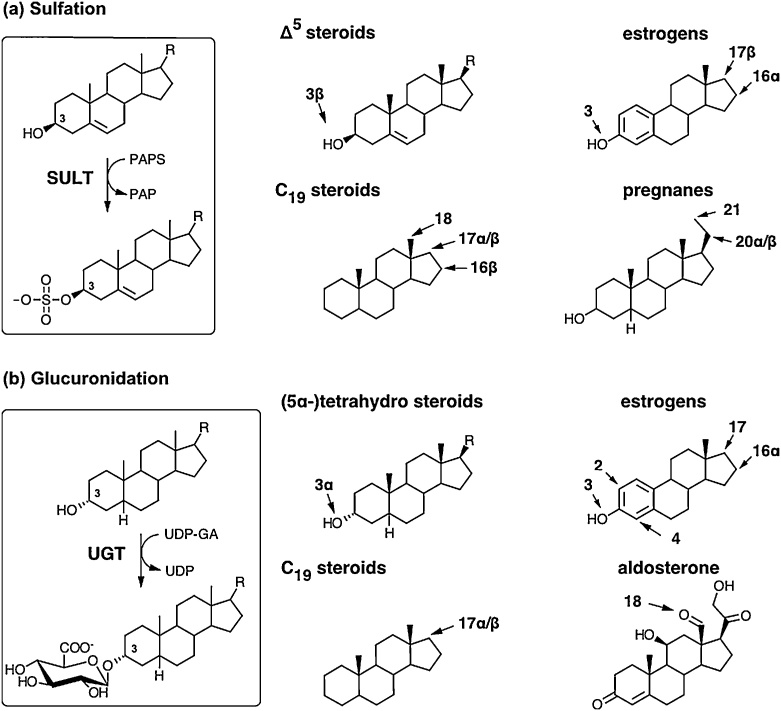
**Schematic overview of the major phase 2 reactions contributing to steroid metabolism – sulfation (a) and glucuronidation (b).** Important target positions of steroid conjugation are indicated, with stereochemistry for the different structural classes of steroids.

Sulfation and desulfation play an essential role in mediating the activity of selected steroids, specifically Δ^5^ steroids and estrogens, as has been comprehensively reviewed recently [40]. Sulfation is the result of two consecutive enzymatic reactions. Firstly, the inert sulfate anion is activated by conversion to the universal sulfate donor 3′-phospho-adenosine-5′-phosphosulfate (PAPS), which is catalyzed by two human PAPS synthase isoforms, PAPSS1 and PAPSS2 [164]. Secondly, the sulfate moiety is transferred onto hydroxy or amino groups by sulfotransferases (SULTs) whereby stereochemistry is retained (Fig. 5(a)). Sulfation is of particular relevance for Δ^5^ steroids which are almost exclusively excreted as their sulfates. Five cytoplasmic SULTs are involved in the sulfation of steroids in humans: SULT1A1, SULT2E1, SULT2A1 and two isoforms of SULT2B1 (SULT2B1a and SULT2B1b) [40,165]. These SULTs have overlapping substrate spectra, but the major enzymes responsible for the sulfation of selected steroids have been identified and are presented in Section 4 below.

Notably, although the contribution of bis-sulfates to the steroid metabolomes of urine, blood and bile was first established in the 1960s [159,160,[166], [167], [168]], interest in these species has only recently re-emerged with the development of new methodological approaches [161,169].

Importantly, sulfation is reversible and unconjugated bioactive steroids can be regenerated from their sulfates by STS, which is ubiquitously expressed in all tissues [40]. STS activity is upregulated in several steroid-dependent cancers [40] and has been evaluated as drug target [170,171]. The 17α- and 20α-sulfates of steroid bis-sulfates are not substrates for STS [172].

#### 3.2.2 Steroid glucuronidation

Glucuronidation makes a substantial contribution to the phase 2 metabolism of Δ^4^ steroids. UDP-glucuronic acid is the activated donor molecule for glucuronidation. Subsequently, the glucuronic acid is coupled with a steroid hydroxy group leading to the formation of the steroid β-D-glucuronide, whereby the stereochemistry of the steroid in the respective position is preserved (Fig. 5(b)) [173,174]. These reactions are catalyzed by enzymes of the UGT-glucuronosyltransferase superfamily (UGTs), with the UGT1A and UGT2B subfamily catalyzing the glucuronidation of steroids [175]. These UGTs are expressed in the liver, as well as in a range of extrahepatic tissues [176,177]. A-ring reduced steroid metabolites are predominantly excreted as 3-glucuronides. Notably, the formation of linked di-glucuronides and bis-glucuronides is also possible [178].

#### 3.2.3 Methylation of catecholestrogens

The O-methylation of catechols plays an important role in the phase 2 metabolism of estrogens (Section 4.4.1) and is catalyzed by the enzyme catechol-O-methyltransferase (COMT) [179]: COMT methylates 2- and 4-hydroxyestrogens, thereby producing the so-called methoxyestrogens [180]. The donor molecule for the methyltransfer is S-adenosylmethionine, which is synthesized from methionine and ATP. COMT primarily methylates the 2 or 4 position of the catechol substrate [181,182]. The highest levels of COMT are found in the liver, brain, kidney, adrenal and lungs [183]. While methylation plays an important role in inactivating catecholestrogens, this phase 2 conjugation reduces water solubility as opposed to the classic phase 2 reactions described above [91].

#### 3.2.4 Steroid thioether formation

Cysteine conjugates of androgens, cortisol and progesterone have recently been detected in human urine and plasma [[184], [185], [186]]. The authors proposed a metabolic pathway starting with a dehydrogenation of the steroid in the liver [187], followed by glutathione S-conjugation of the steroid and subsequent extracellular degradation of the glutathione moiety leading to a cysteine conjugate which is excreted.

#### 3.2.5 Fatty acid esterification of steroids

Although the physiological relevance has yet to be determined, fatty acid esterification of pregnenolone, DHEA and androstenediol has been described [188,189]. Plasma lecithin:cholesterol acyltransferase located on high-density lipoproteins can acylate steroids using acyl-CoA as donor [190]. The steroid fatty acid ester can then be transferred to other lipoproteins and be taken up by peripheral cells via lipoprotein receptors [191,192]. Additionally, steroids can be esterified with fatty acids in peripheral tissues [193].

### 3.3 Steroid excretion

Steroids are excreted predominantly as their conjugates in the urine and bile, with urine excretion accounting for approximately 80% of excretion following exogenous administration [194]. The clearance of steroid glucuronides generally proceeds faster than the clearance of steroid sulfates [195], presumably due to the irreversible nature of glucuronidation in humans.

#### 3.3.1 Urinary steroid excretion

In the kidney, steroid conjugates are transported from the blood filtrate over the epithelium into the lumen of the nephron. Cellular uptake is mediated by organic anion symport or exchange. The efflux from the cell into the lumen is carrier-mediated and makes use of an electrochemical gradient [196]. The active nature of transport across the epithelial cells allows for the concentration of steroid conjugates in the lumen. Urinary excretion of unconjugated steroids is low, accounting for only 5–10% of the total urine steroid pool. The urine metabolomes of all classes of steroids will be discussed in detail in Section 4. Interestingly, enzymes with 20β-HSD and 17,20-lyase activity have been recently identified in a microbial inhabitant of the urinary tract [197].

#### 3.3.2 Biliary excretion of steroids

Steroids passing the canalicular membrane of the hepatocyte are excreted in the bile. The rate of biliary excretion determines the quantitative contribution of the gut microbiome to the metabolism of the respective steroid. Excretion with the feces is low as steroids are reabsorbed in the gut [198]. 17-deoxysteroids (mineralocorticoids and precursors) have high biliary excretion as opposed to 17-hydroxy C_21_ steroids like cortisol [147]. Steroid conjugates represent the major fraction of biliary excreted steroids [147,[199], [200], [201], [202]] and the dominance of bis-sulfates [168] led to the hypothesis that bis-sulfates originating from the liver are preferably excreted with the bile, while glucuronides preferably undergo renal excretion [200]. Biliary excretion is increased during pregnancy [201,203,204] and an equal quantitative contribution of urinary and fecal excretion has been suggested during the newborn period [204]. The steroid metabolome in the feces comprises unconjugated, mono- and bis-sulfated steroids [203] and up to 90% of steroids in feces during pregnancy are unconjugated [205]. Notably, estrogens seem to undergo higher biliary excretion than other steroids, but also higher reabsorption leading to low fecal excretion [194,206]. Gut microbiota have hydrolase activity for steroid conjugates and glucuronides in particular [148], leading to the high proportion of unconjugated steroids compared to bile, and conjugate hydrolysis might be a prerequisite for reabsorption [207].

#### 3.3.3 Salivary steroids

Steroids are also excreted in saliva. Indeed, the measurement of salivary steroids is becoming an emerging tool for the diagnosis and treatment monitoring of steroidogenic disorders due to the ease of saliva collection [208]. Unconjugated steroids passively diffuse over the membranes of the acinar cells in the salivary glands independent of salivary flow rate [209]. Their levels in saliva therefore provide a measure of their free concentrations in serum [210]. However, steroids can be subject to metabolism while crossing the acinar cells, which affects their levels in saliva. For example, the presence of HSD11B2 in the parotid gland makes salivary cortisone a useful marker for serum free cortisol and adrenal stimulation [[211], [212], [213]]. Conjugated steroids enter saliva by ultrafiltration through the extracellular space between the acinar cells and their salivary levels are highly flow rate dependent as has been shown for DHEAS [209].

## 4 Serum and urine steroid metabolomes

### 4.1 The mineralocorticoid metabolome

The primary mineralocorticoid in circulation is aldosterone. It is estimated that approximately one third of aldosterone circulates in the free form, with the remainder bound to corticosteroid binding globulin (CBG) and serum albumin [214]. Serum mineralocorticoid assays primarily focus on the measurement of aldosterone only, although the precursors DOC, corticosterone and 18-hydroxycorticosterone are also detectable in serum (Table 2) [215].

**Fig. 6:**
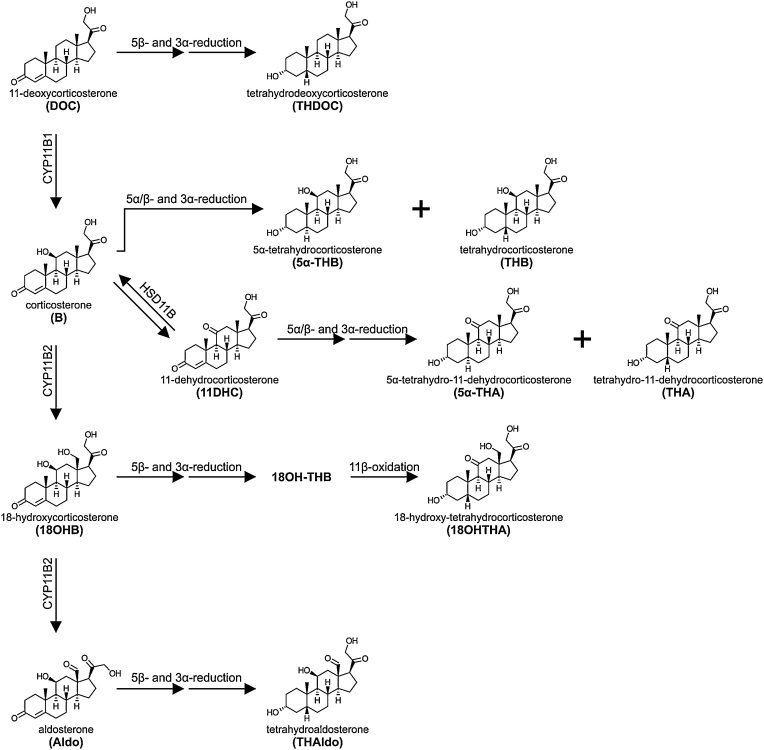
**Schematic overview of the pathways linking mineralocorticoids and their precursors to their urine metabolites.** The pathway of mineralocorticoid biosynthesis is indicated on the left. The metabolism of each steroid is shown from left to right and the structures of the major urine products are shown. Phase 2 conjugation reactions are not indicated in the figure.

Aldosterone, as well as its immediate precursors, contain a Δ^4^ moiety. Metabolism of these steroids is therefore primarily by sequential 5α/β- and 3α-reductions in the liver (Fig. 6). DOC and aldosterone are both preferentially 5β-reduced by AKR1D1 followed by 3α-reduction yielding the tetrahydro metabolites, tetrahydrodeoxycorticosterone (THDOC) and tetrahydroaldosterone (THAldo), respectively. Similarly, 18-hydroxycorticosterone is converted to the tetrahydro metabolite, 18-hydroxy-tetrahydro-11-dehydrocorticosterone (18OHTHA), but this requires the additional conversion of the 11β-hydroxy group to an 11-keto group, a reaction catalyzed by HSD11B2 [[216], [217], [218]]. Corticosterone can be converted to 11-dehydrocorticosterone by the action of HSD11B2 in the kidney, which prevents the activation of the mineralocorticoid receptor (MR) by corticosterone though corticosterone’s MR-activating potency is considerably lower than that of aldosterone [219]. Unlike the other mineralocorticoid precursors, both corticosterone and 11-dehydrocorticosterone can either be 5α- or 5β-reduced, prior to 3α-reduction leading to a tetrahydro and a 5α-tetrahydro metabolite for each. These are tetrahydro-11-dehydrocorticosterone (THA) and 5α-tetrahydro-11-dehydrocorticosterone (5α-THA) for 11-dehydrocorticosterone, and tetrahydrocorticosterone (THB) and 5α-tetrahydrocorticosterone (5α-THB) for corticosterone, the latter being dominant (Fig. 6). Importantly, during neonatal life, THA, 5α-THA and the polar metabolite 6α-hydroxy-THA are the more relevant corticosterone metabolites (Fig. 4(g)) [130]. As with most steroid metabolites, the majority of these are subsequently glucuronidated in the liver and excreted in urine [[220], [221], [222]]. Aldosterone is preferentially 18-glucuronidated in the liver by UGT2B7 and UGT1A10 [223]. UGT2B7 also efficiently conjugates 5α-dihydroaldosterone and THAldo, while UGT2B4 glucuronidates only THAldo [224]. It should, however, be noted that aldosterone is also glucuronidated directly to aldosterone-18β-glucuronide within the kidney, which has been proposed to be catalyzed by UGT2B7 [225]. Indeed, it has been estimated that aldosterone-18β-glucuronide and THAldo-glucuronides contribute 5–15% and 15–40% towards the daily urinary excretion of aldosterone [221,226]. It has also been found that THAldo-glucuronides are consistently five-fold more prevalent than aldosterone-18β-glucuronide irrespective of sodium intake [221]. Finally, it should be noted that enzymes expressed by anaerobic bacteria in the human gut have been shown to be able to convert aldosterone to THAldo, 3β,5α-THAldo, 3α,5α-THAldo as well as 20β-dihydroaldosterone in a species-specific manner [150]. Biliary excretion and metabolism by the gut microbiome is also relevant for the 17-deoxy mineralocorticoid precursors and their metabolites as described in Section 3.1.3.4 [227].

### 4.2 The glucocorticoid metabolome

#### 4.2.1 Cortisol and cortisone interconversion

The primary active glucocorticoid in circulation is cortisol, which is produced by the adrenal cortex (Section 2.5.2). Inactivation of cortisol to cortisone, which cannot activate the glucocorticoid receptor or the MR, subsequently occurs in peripheral mineralocorticoid target tissue, such as the kidney, the colon and the salivary glands all of which express HSD11B2 [74]. HSD11B2 converts cortisol to cortisone to protect the MR from activation by cortisol, thus allowing the dedicated MR agonist aldosterone to bind [[228], [229], [230], [231]]. The placenta is another important tissue expressing HSD11B2 as to inactivate maternal cortisol, thereby limiting fetal exposure [74].

Cortisone is in-turn reactivated to cortisol by the action of HSD11B1 expressed predominantly in the liver, as well as some peripheral tissues such as adipose tissue, muscle, skin and bone [74,[232], [233], [234], [235]]. Although the ratio of cortisol to cortisone remains relatively constant in circulation, studies with radiolabeled tracers have shown that there is constant interconversion of cortisol and cortisone [236]. Tissue-specific expression of HSD11B1 allows for local intracellular cortisol reactivation independently of circulating cortisol levels [74]. Interestingly, HSD11B1 expression is low/undetectable at birth, but thereafter increases rapidly, with adult levels reached after 6–12 months [237]. As a result, cortisone and the resulting 11-keto-metabolites (e.g. tetrahydrocortisone) are substantially increased during the neonatal period [74].

While HSD11B1 can function bi-directionally *in vitro*, it acts predominantly as a reductase *in vivo* due to localized co-expression on the ER membrane with H6PDH, which produces NADPH that drives the reductase activity of HSD11B1. A deficiency of H6PDH therefore leads to an impairment of HSD11B1 reductive function and apparent cortisone reductase deficiency [238].

A vital aspect to consider when measuring cortisol and cortisone is their diurnal secretion rhythm [239]. This follows a distinct pattern with nadir concentrations around midnight and the highest levels observed between 3 and 5 a.m., although the exact timings can show inter-individual variability [[240], [241], [242]]. Any healthy individual’s serum cortisol concentration will be significantly higher in the morning than at midnight. This rhythm can be lost in times of severe illness or stress, with significant increases in circulating cortisol throughout the entire 24-h period observed in patients with sepsis and during surgical procedures [243,244]. To account for the diurnal rhythm of glucocorticoids, when assessing serum glucocorticoid concentrations, comparator samples should be drawn at the same time of day. With urine collections, the time of collection is similarly important – a one-off spot urine collected in the morning will differ greatly from a spot urine collected in the afternoon, a problem that can be overcome by 24-h urine collections, which provide output data for the entire 24-h period independent of diurnal variation.

The majority of cortisol circulates bound to proteins, with 80–90% bound to CBG. CBG also binds other steroids such as cortisone, 17OHP, progesterone, DOC, corticosterone, and, to a lesser degree, aldosterone, testosterone and 17β-estradiol. The remaining cortisol is either bound to albumin (5–10%) or circulates in its free (active) form (<10%) [[245], [246], [247]].

The serum levels of the glucocorticoid precursors, progesterone, 17OHP and 11-deoxycortisol are all ≤10nM in the healthy population and therefore significantly lower than that of cortisol (ranging from 100 to 600 nM) and cortisone (ranging from 30 to 100 nM) (Table 2). The concentrations of cortisol, cortisone and 11-deoxycortisol are similar in men and women, though women who have increased estrogenlevels due to oral or transdermal contraceptives or pregnancy, have increased total serum cortisol due to an increase in CBG (and in pregnancy, also an increase in total cortisol production from the 22nd week of gestation onwards) [[248], [249], [250]].

#### 4.2.2 Downstream glucocorticoid metabolism

**Fig. 7:**
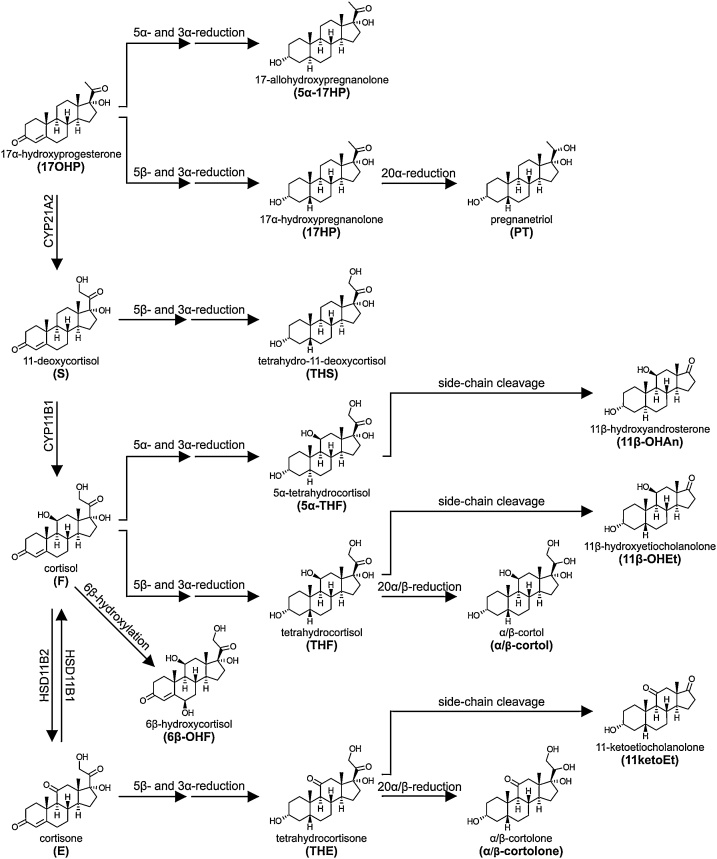
**Schematic overview of the pathways linking glucocorticoids and their precursors to their urine metabolites.** The glucocorticoid biosynthetic pathway is shown on the left. The metabolism of each steroid is shown from left to right and the structures of the major urine products are shown. Phase 2 conjugation reactions are not indicated in the figure.

As with all steroids containing the Δ^4^ steroid moiety, the dominant first steps in the metabolism of glucocorticoids is the 5α/5β-reduction in the liver (Section 3.1.1). Interestingly, while 17OHP and cortisol can be either 5α- or 5β-reduced, 11-deoxycortisol and cortisone are predominantly 5β-reduced [108]. All products are subsequently 3α-reduced yielding the respective tetrahydro or 5α-tetrahydro metabolites (Fig. 7), which are detectable in urine. A number of the tetrahydro metabolites can also be further metabolized by 20α- or 20β-HSDs [251,252]. Of quantitative importance is the 20α/β-reduction of tetrahydrocortisol (THF), yielding the so called cortols (α- and β-cortol), and that of tetrahydrocortisone (THE), which yields the equivalent cortolones (α- and β-cortolone) [253]. While all four members of the human AKR1C enzyme subfamily can catalyze the reduction to the 20α-hydroxy group, AKR1C1 is the predominant 20-ketosteroid reductase in human [104]. Although a 20β-HSD reducing cortisone in zebrafish has been characterized as a member of the SDR family [254] and carbonyl reductase 1 has been described a relevant 20β-HSD for cortisol in humans [255], the human enzyme(s) responsible for the formation of the 20β-hydroxy isomers of the cortols and cortolones has not yet been identified. Cortisone and cortisol reduced at 20α- and 20β- while retaining the Δ^4^ moiety are also excreted in significant amounts, i.e. 20α(andβ)-dihydrocortisone and 20α-(andβ)-dihydrocortisol [256].

THF and THE can also be subject to an elusive side-chain cleavage reaction not catalyzed by CYP17A1, producing C_19_ metabolites of glucocorticoid origin. Thereby, glucocorticoids contribute predominantly to urinary excretion of 11β-hydroxyetiocholanolone and 11-ketoetiocholanolone. While 5α-THF is also a substrate for this side-chain cleavage reaction, this reaction is catalyzed less efficiently [257]. As a result, the glucocorticoid contribution to urine 11β-OHAn is very low in healthy individuals, with the majority originating from the metabolism of the androgen 11OHA4 (Section 4.3.2) [258].

Cortisol can also be 6β-hydroxylated by CYP3A4 expressed in the liver, resulting in 6β-hydroxycortisol (6β-OHF) [259,260]. Orally administered hydrocortisone results in relatively increased circulating 6β-OHF, in comparison to the other glucocorticoid metabolites, due to the hepatic first pass effect after the oral ingestion. 18-Hydroxycortisol is also a product formed in *zona fasciculata* of the adrenal, with the minor downstream product 18-oxocortisol also being produced. These metabolites are often referred to as “hybrid steroids” as they require enzymatic machinery from both the glucocorticoid and mineralocorticoid pathways [[261], [262], [263]]. They are particularly important in patients in glucocorticoid remediable aldosteronism or with aldosterone-producing adenomas associated with *KCNJ5* mutations [261,[264], [265], [266], [267], [268], [269], [270], [271], [272]]. Hydroxylation of the tetra- and hexahydro-metabolites of cortisol (e.g., THE and the cortolones) at 1β- and 6α-carbon position is quantitatively important in the neonatal period. [130]. The glucocorticoid precursor 17OHP can be converted to 17α-hydroxypregnanolone (17HP) via 5β-reductase and 3αHSD activities. The subsequent 20α-reduction of 17HP yields the metabolite pregnanetriol (PT, 5β-pregnane-3α,17α,20α-triol).

The majority (>90%) of the glucocorticoid metabolites described above are glucuronidated in the liver prior to urinary excretion as mono-glucuronides with glycosidic bonds added at positions 3 or 21. UGT2B7 has been shown to efficiently catalyze the conjugation of glucocorticoids [224]. Metabolites retaining the Δ^4^ moiety are excreted to a greater degree unconjugated. Two studies report unconjugated excretion of the following individual steroids: cortisol (30%), cortisone, 20αDHE, 20βDHE, 20βDHF (40–60%); 20αDHF, 6β-OH-cortisol, 6β-OH-E and 18-OH-cortisol (80–100%) [273,274]. Urine free cortisol, i.e. the free fraction of total, non-metabolized urine cortisol, is commonly measured in clinical chemistry laboratories for the diagnosis of Cushing’s syndrome [275] whereas gas chromatography-mass spectrometry (GC-MS) measures total urine cortisol following deconjugation.

### 4.3 The androgen metabolome

#### 4.3.1 Androgens in circulation

Androgens and their precursors are derived from both the adrenal cortex and the gonads as described in Section 2.5.3 above. It is important to note the that circulating androgen metabolome consist of both active androgens and androgen precursors, with both of these contributing to androgen action in target tissues [45]. Downstream metabolites can also be measured in circulation [60]. The best-known circulating androgen in both men and women of reproductive age is testosterone (Table 2). Circulating testosterone concentrations in men are approximately 10-fold higher than those of women, due to the dedicated biosynthesis in the testes, together with a very minor contribution from the adrenals (Section 2.5.3.1). Conversely, female androgens are equally derived from the adrenal glands and the ovaries (Section 2.5.3.1), which are each estimated to contribute 25% towards the circulating levels of testosterone in both pre- and postmenopausal women. The remaining 50% originates from the peripheral conversion of androgen precursors such as A4 to testosterone [[276], [277], [278]]. Androgen precursors in circulation include DHEA, its sulfate ester DHEAS, A4, 11OHA4, androstenediol and androstenediol sulfate. In fact, the circulating levels of DHEAS dwarf those of any other steroid in circulation (Table 2) and DHEAS is the only human steroid that circulates in micromolar concentrations. However, it is primarily thought to serve as an inactive waste product of adrenal steroidogenesis, produced to prevent an excessive androgen load [39,40,279]. The production of adrenal androgen precursors increases at adrenarche at 6–9 years of age, peak between 20 to 30 years of age, and subsequently decline gradually with age (Section 2.5.3.1). Gonadal androgen production is initiated for a short period of time during minipuberty in infancy, but then remains dormant until puberty. Following full initiation at puberty gonadal androgen production decreases significantly after menopause in women, while in men the testicular output of testosterone gradually decreases with age, resulting in significantly lower combined androgen levels in men aged 60 and over [24,[280], [281], [282]]. In women, testosterone and A4 demonstrate cyclic changes in concentration during the course of the menstrual cycle due to the ovarian contribution, with levels peaking mid-cycle [283,284].

Within circulation, most active sex steroids are bound to the plasma proteins sex hormone binding globulin (SHBG) or albumin and only a small fraction (1–2%) circulates unbound, which is the only form in which testosterone is accessible to the target tissues. Sex steroid-binding plasma proteins, therefore, play a key role in the regulation of androgen action. SHBG binds sex steroids (including active androgens and estrogens) with high affinity (nanomolar ranges) and specificity [[285], [286], [287]]. Although albumin binds all unconjugated steroids with low affinities (micromolar ranges), it makes a significant contribution to steroid binding due to its high abundance [45,287]. While SHBG binds the active androgens DHT and testosterone with high affinity, the affinity of SHBG for androgen precursors such as DHEA is substantially lower. Moreover, the conjugated precursor, DHEAS, circulates only in its free form.

While the levels of 11-oxygenated androgens and their precursors have been shown to be significantly elevated in patients with polycystic ovary syndrome (PCOS) and 21-hydroxylase deficiency [41,69], one study reported that the circulating levels of 11KT were also higher than that of testosterone in a healthy female control group (BMI 21.2–26.1 kg/m^2^) [41]. This and other recent findings have led some to suggest that 11KT may in fact be the most physiologically relevant androgen in women, though more work is needed to investigate this [288]. However, although multiple studies have measured the circulating concentrations of 11-oxygenated androgens in healthy control groups, there are significant variations in the levels reported and as such, no reference ranges have been established to date [39,41,68,69,289]. Nonetheless, it is clear that 11OHA4, the major 11-oxygenated androgen precursor produced by the adrenal, circulates at higher levels than A4. 11KA4 is the next most abundant 11-oxygenated androgen precursor in circulation (Table 2) [39,41,68,69,289]. Significantly, a recent study has revealed that unlike the classical androgens, the circulating levels of 11-oxygenated androgens do not decrease with age in women, suggested to be due to the involution of the *zona reticularis* with age and the appearance of areas co-expressing HSD3B2 and CYB5A [289].

#### 4.3.2 Downstream androgen metabolism

The contributions of androgen precursors of adrenal and gonadal origin are often overlooked when considering the total androgen pool. The primary reason for this is that, while androgen precursors are activated in peripheral target tissues, this is often, but not always, followed by subsequent inactivation within the same tissue, thus with the result that much of the active androgen is never accounted for in circulation (Section 2.5.3.2). It is therefore important to consider both androgen precursors and metabolites when accessing androgen action. While androgen precursors and active androgens can be measured in serum, it is often more convenient to measure their metabolites in urine (Table 3).

**Table 3:** Graphical representation of the targeted urine steroid metabolome. Major urine steroids are shown divided into six concentration ranges illustrating their relative contribution to total 24 h urine steroid metabolite excretion. Divisions are based on respective median values as urine metabolites demonstrate substantial variation between individuals and the 25-75^th^ percentiles may overlap groups.

					
1000-3000 μg/24 h	700-1000 μg/24 h	400-700 μg/24 h	150-400 μg/24 h	20-150 μg/24 h	<20 μg/24 h
**5α-THF** (male)	**5α-THF** (female)	**β-cortol** (male)	**17HP** (male)	**17HP** (female)	**PTONE**
**THF**	**α-cortolone** (female)	**PT** (male)	**PD**	**5 P T** (female)	**THDOC**
**α-cortolone** (male)	**Et** (female)	**11β-OHAn** (male)	**5 P T** (male)	**THS**	**5α-17HP**
	**An** (female)		**PT** (female)	**THAldo**	
**THE**			**5α-THB**	**18OHTHA**	
**An** (male)			**α-cortol**	**THA**	
**Et** (male)			**11β-OHEt**	**5α-THA**	
			**β-cortol** (female)	**THB**	
			**11ketoEt**	**cortisol**	
			**β-cortolone**	**cortisone**	
			**DHEA**	**6β-OHF**	
			**16α-DHEA** **11β-OHAn** (female)		

**Fig. 8:**
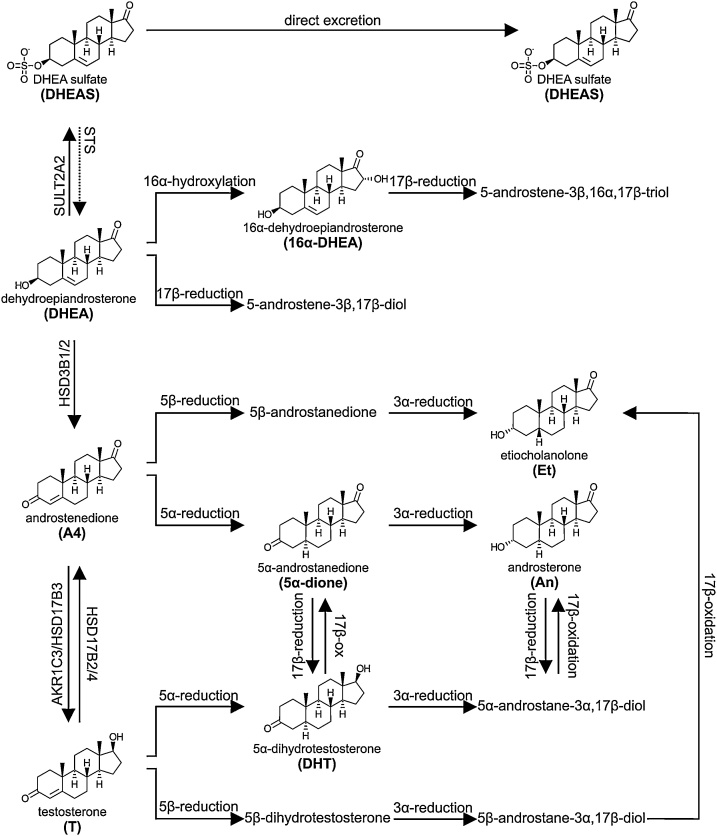
**Schematic overview of the pathways linking androgens and their precursors to their urine metabolites.** Major serum androgen precursors and androgens are shown on the left. The metabolism of each steroid is shown from left to right and the structures of the major urine products are shown. 5α-dihydrotestosterone (DHT), the most potent androgen, is derived from testosterone by 5α-reduction and, thus, its formation is only reflected by urine androsterone. Phase 2 conjugation reactions are not indicated in the figure.

Undoubtedly, the most important step in androgen activation and inactivation is the 5α-/5β-reduction of the Δ^4^ steroid moiety common to all androgen precursors as well as the potent androgen testosterone (Fig. 8). This moiety is selectively 5α-reduced by the action of steroid 5α-reductase enzymes within target tissues. Those androgens and precursors that escape the tissue specific activation via 5α-reduction are metabolized within the liver, which expresses both 5α- and 5β-reductases [54,94,100]. Unlike 5α-reduction, which is required to produce the potent androgen DHT, AKR1D1-catalyzed 5β-reduction acts only as an inactivation step. Even 5β-DHT, the product of the 5β-reduction of testosterone, is an inactive androgen metabolite [54]. Following 5α/5β-reduction, androgen metabolites are subject to reduction of the 3-keto group with predominant 3α-stereoselectivity [54]. Importantly, 3α,5α-reduced metabolites can potentially be converted back to the 3-keto metabolite by oxidative 3α-HSDs such as in the alternative DHT biosynthesis pathway (Section 2.5.3.3).

The majority of 5α/β-3α-metabolites of testosterone and DHT, which contain a 17β-hydroxy, are converted to 17-keto steroids by the action of the oxidative 17β-HSDs, HSD17B2 and HSD17B4 [106]. As a result, androgen metabolites are excreted with a 17-keto/17β-hydroxy ratio of approximately 10:1 [112].

Therefore, the primary urine androgen metabolites are androsterone (An; 5α-androstan-3α-ol-17-one) and etiocholanolone (Et; 5β-androstan-3α-ol-17-one) (Table 3). While, A4 and testosterone can be metabolized to either androsterone or etiocholanolone, DHT is 5α-reduced and thus only reflected in the androsterone fraction.

Both An and Et are subject to glucuronidation at the 3 position. This phase 2 metabolism can occur in the liver or within peripheral target tissues. The glucuronidation of C_19_ steroids is catalyzed by three members of the UGT2B subfamily, namely: UGT2B7, UGT2B15 and UGT2B17 [175]. The three enzymes have differential regioselectivity and substrate specificity for the 5α/β-stereoisomers [290]. UGT2B7 glucuronidates only the hydroxy group at position 3, but not in position 17 and preferentially conjugates 5α- over 5β-androstanes. UGT2B7 is the most efficient UGT for androstanediol conjugation [176]. UGT2B15 does not target the 3-hydroxy group, but conjugates the 17-hydroxy group in the androstane-3α,17β-diols, such as testosterone or DHT, and prefers the 5α-stereoisomers. Similarly, UGT2B17 has a preference for the 17β-hydroxy group in the androstane-3α,17β-diols, but conjugates the 3α-hydroxy group of An and Et with Et being the preferred substrate [290]. UGT2B17 has highest activity of all UGTs towards An, testosterone and DHT. UGT2A1 may also contribute to the glucuronidation of testosterone [291]. Interestingly, UGT2B15 which is expressed in adipose tissue has been shown to demonstrate a higher activity in obese individuals, which may contribute to the increased levels of 3α-androstanediol glucuronide observed in obesity [292,293]. While Δ^5^ steroids like DHEA and pregnenolone are excreted almost exclusively as sulfates, sulfation of other C_19_ steroid metabolites are considered minor phase 2 reactions. SULT2A2 can target 3α- and 17β-hydroxyl groups and has been shown to sulfate An, testosterone and DHT [294,295]. Hydroxy groups in positions 16β, 17α/β and 18 are also important targets for sulfation of C_19_ steroids [159,[296], [297], [298]].

Major urine androgen precursor metabolites include DHEA and 16α-hydroxy-DHEA. Circulating DHEA is readily 16α-hydroxylated by CYP3A4/7 within the liver [136,299]. The abundant conjugated androgen precursor, DHEAS, is water-soluble and is largely excreted in an unmodified form as represented by the urinary DHEA fraction following deconjugation.

Urinary metabolite excretion deriving from the 11-oxygenated androgen precursor 11OHA4 is well understood. 11OHA4 undergoes sequential 5α- and 3α- reduction yielding 11β-OHAn, which is readily quantifiable in urine (Table 3). It should be noted that although 11β-OHAn can also derive from cortisol metabolism (Section 4.2.2), this only contributes to approximately 5–10% of the measured levels, with at least 90% originating from 11OHA4 [258]. The metabolism of the active 11-oxygenated androgen, 11KT, has yet to be fully elucidated. Similarly, only a few studies have investigated the potential conjugation of 11-oxygenated steroids. While these steroids do appear to be glucuronidated, the limited data at hand suggests that glucuronidation of these steroids is less efficient than what is observed for the classic androgens [300].

### 4.4 The estrogen and progestogen metabolomes

#### 4.4.1 The estrogen metabolome

The primary estrogens in circulation are estrone, estrone sulfate and 17β-estradiol, with 17β-estradiol considered the biologically active form [82,[301], [302], [303]]. In premenopausal women, these estrogens are predominantly produced by the ovaries (Section 2.5.4), but estrogens are also synthesized in peripheral tissues expressing aromatase, such as adipose tissue, using adrenal-derived androgen precursors. This peripheral production of estrogens is especially important in postmenopausal women and men [61]. It should be noted that this peripheral estrogen production often functions in a paracrine and intracrine manner and as such circulating concentrations are not reflective of the concentrations achieved locally [304,305]. Circulating levels of estrogens vary greatly during the course of the menstrual cycle and decrease significantly in postmenopausal women (Table 3) [[306], [307], [308]]. Notably, estrone sulfate is the predominant estrogen in circulation for both men and premenopausal women and serves as a biologically inactive reservoir for the generation of active estrogens in target tissues [40,309,310]. Like with androgens, the majority of unconjugated estrogen circulates bound to SHBG with high affinity and albumin with low affinity [[285], [286], [287]]. Another similarity to androgens is the regulation of estrogen potency by HSD17B enzymes, with HSD17B1 and HSD17B2 being the two most prominent isoforms involved in estrogen metabolism. HSD17B1 reduces estrone to the most active estrogen, 17β-estradiol. HSD17B2 catalyzes the reverse oxidative reaction of 17β-estradiol to estrone in addition to its high activity towards androgens. Further metabolism of both estrone and 17β-estradiol can yield estriol. Estrone undergoes 16α-hydroxylation and HSD17B1 catalyzed reduction, while 17β-estradiol only requires 16α-hydroxylation [[311], [312], [313], [314]]. CYP3A4 is the major enzyme responsible for the 16α-hydroxylation of estrone in adults, though CYP1A1, CYP2C19 and CYP3A5 can also catalyze the reaction [315,316]. Conversely, CYP1A2 is the dominant enzyme catalyzing the 16α-hydroxylation of 17β-estradiol, with CYP3A4, CYP1A1 and CYP1B1 also demonstrating this activity [317]. Estriol is rapidly excreted in urine and, as a result, serum levels are low to undetectable [318].

Both estrone and 17β-estradiol can also undergo hydroxylation at position 2 and 4 [141,317,[319], [320], [321]]. These reactions are catalyzed by a variety of CYPs, including CYP3A4 and CYP1A2 in the liver, or CYP1A1 and CYP3A4 in peripheral tissues. In the liver approximately 80% of 17β-estradiol is hydroxylated to the 2 position and 20% at the 4 position [322]. 2- and 4-hydroxy groups on the A-ring can be methylated as introduced in Section 3.2.3. Other reported hydroxylations include those at 6α, 6β, 7α, 12β, 15α, 15β, 16α and 16β positions as well as further oxidation to a 6-ketone or 9-11-dehydrogenation [141,[322], [323], [324]].

Estrogens and catecholestrogens are efficiently sulfated at several positions [[325], [326], [327]]. SULT1E1 is the major SULT for estrogen sulfation [328,329], while SULT1A1 and SULT1A3 also sulfate estrogens, but with a lower affinity [327].

Glucuronidation of estrogens is catalyzed by members of the UGT1A and UGT2B7 subfamilies with the UGT1A isoforms making the largest contribution to the glucuronidation of estrone and 17β-estradiol. Estriol and 16α-hydroxyestrone are conjugated at the 3-hydroxygroup by UGT1A10 and at the 16α-hydroxy group by UGT2B7 [330,331]. Catcholestrogens can additionally be glucuronidated in positions 2 and 4 [332].

#### 4.4.2 The progestogen metabolome

Progestogens are compounds with progestational activity, referring to their induction of a secretory endometrium to support gestation [333]. The only true natural progestogen is progesterone. Levels change substantially during the course of the menstrual cycle, peaking during the luteal phase (Table 3). Low levels of circulating progesterone are also detectable in men [334]. Progesterone primarily circulates bound to CBG. During the second and third trimesters of pregnancy placental trophoblasts produce large amounts of progesterone, which displaces glucocorticoids from CBG [287,335].

Progestogens are primarily metabolized by the liver largely to form pregnanediols and pregnanolones [336,337]. Progesterone is metabolized to pregnanediol (PD, 5β-pregnane-3α,20α-diol) in three steps. AKR1D1 catalyzes the 5β-reduction followed by members of the AKR1C enzyme family catalyzing subsequent 3α- and 20α-reductions. Alternatively, progesterone can first be reduced to 20α-hydroxyprogesterone, which can then be further 5β-reduced by AKR1D1 and 3α-reduced by AKR1C1–4 [103]. PD is efficiently glucuronidated at position 3, resulting in pregnanediol-3-glucuronide being the major progesterone metabolite identified in urine. Progesterone metabolites reduced at 5α position are subject to extrahepatic 6α-hydroxylation, which is distinct from the hepatic 6α-hydroxylation active on Δ^4^ steroids [338].

## 5 Steroid metabolome profiling by mass spectrometry

### 5.1 Current state-of-the-art techniques in steroid analysis

Mass spectrometry is a powerful technique with which multiple steroids can be measured within a single analytical run. Despite the wealth of information that can be achieved by these methods, uptake in the clinical setting is still limited, primarily due to the cost of the technology and the limited availability of the required expertise.

Currently, gas chromatography-mass spectrometry (GC-MS) is the preferred method for the analysis of urine steroids in research laboratories due to the unparalleled resolution offered by this technique [339,340]. However, of late, there are increasing efforts to develop both ultra-high performance liquid chromatography-tandem mass spectrometry (UHPLC-MS/MS) and ultra-high performance supercritical fluid chromatography-tandem mass spectrometry (UPHSFC-MS/MS) methods for the screening of multiple urine steroids [341,342]. An advantage of these techniques is that deconjugation is not mandatory, unlike with GC—MS. The idea of quantifying conjugated urine steroid metabolites is therefore gaining momentum in the field. This may be advantageous as some steroids with secondary sulfate groups (bis-sulfates), or glucuronides can be resistant to common hydrolysis procedures.

The introduction of high throughput UHPLC-MS/MS has led to a substantial increase in the use of mass spectrometry-based assays for steroid profiling, especially in serum, as UHPLC-MS/MS is a more accurate and reliable technique without the cross-reactivity issues that plague immuno-based assays. Indeed, there is a drive within the endocrine community to phase out immunoassays where possible [343]. Moreover, the use of high-resolution accurate mass (HRAM) mass spectrometry coupled to liquid chromatography systems is being explored as an alternative to traditional MS/MS systems as accurate mass quantification offers the potential to resolve all steroid metabolites with the exception of steroid isomers, unless they are separated chromatographically [344].

It should, however, be noted that despite the advantages of mass spectrometry techniques, these are not without their challenges. Perhaps the biggest challenge to the endocrine community is the cross validation of methodologies employed in different laboratories. Currently differences in sample work-up methodologies and/or instrumentation and settings can result in reference ranges that vary between laboratories. Moving forward methods therefore ideally need to be validated both internally according to set standards and subsequently compared using standardized reference material and quality controls [[345], [346], [347]].

### 5.2 Steroid metabolomics

Steroid metabolomics is defined as the combination of steroid metabolome profiling by mass spectrometry with computational machine learning-based analysis of the mass spectrometry data. Such sophisticated and unbiased computational analysis techniques have shown potential for assisting and even automating analysis of large or highly heterogeneous datasets, making it an ideal resource for use in metabolomics. Machine learning involves training a computer program to recognize patterns within large-scale data - the more data it is exposed to, the greater the learning capability. This generates a tailor-made diagnostic algorithm that can be prospectively applied to newly recorded steroid data. Interpretable models can help to understand underlying mechanisms, categorize and classify, or even make predictions based on observed patterns in the data. As an example, this approach has been used for automating differentiation of adrenocortical carcinoma (ACC) from benign adrenocortical tumors based on the detection of a “malignant steroid fingerprint”, a distinct set of urine steroid metabolites characteristically increased in ACC [348]. The principle established in this example has opened the door for the application of this approach to other steroidogenic disorders that create a unique steroid “fingerprint”.

## 6 Conclusion

The biosynthesis and metabolism of steroid hormones is complex. Although the measurement of individual steroids has routinely been employed for the diagnosis of endocrine conditions for many years, advances in technology now allow for the high throughput profiling of comprehensive steroid panels, thereby offering significantly more information and diagnostic power. Furthermore, the use of unbiased computational approaches such as machine learning allows for the development and implementation of steroid metabolomics analysis, which has the potential to not only improve, accelerate and automate diagnostics, but also to lead to improvements in treatment monitoring and prognostic prediction. Nonetheless, a detailed understanding of steroid biosynthesis and the principles that govern steroid metabolism and excretion remains fundamental to the accurate interpretation of metabolomics data as well as the improvement of our understanding of associated disorders.

## Declaration of Competing Interest

W.A. is an inventor on a patent for the use of steroid profiling as a biomarker tool in the differential diagnosis of steroid-producing and steroid-dependent tumors (PCT/GB2010/000274). All other authors did not declare a conflict of interest.
